# SARS-CoV-2 infection triggers paracrine senescence and leads to a sustained senescence-associated inflammatory response

**DOI:** 10.1038/s43587-022-00170-7

**Published:** 2022-01-25

**Authors:** Shunya Tsuji, Shohei Minami, Rina Hashimoto, Yusuke Konishi, Tatsuya Suzuki, Tamae Kondo, Miwa Sasai, Shiho Torii, Chikako Ono, Shintaro Shichinohe, Shintaro Sato, Masahiro Wakita, Shintaro Okumura, Sosuke Nakano, Tatsuyuki Matsudaira, Tomonori Matsumoto, Shimpei Kawamoto, Masahiro Yamamoto, Tokiko Watanabe, Yoshiharu Matsuura, Kazuo Takayama, Takeshi Kobayashi, Toru Okamoto, Eiji Hara

**Affiliations:** 1 Department of Molecular Microbiology, Research Institute for Microbial Diseases, Osaka University, Suita, Japan; 2 Department of Virology, Research Institute for Microbial Diseases, Osaka University, Suita, Japan; 3 Center for iPS Cell Research and Application, Kyoto University, Kyoto, Japan; 4 Division of Infectious Diseases, Institute for Advanced Co-Creation Studies, Osaka University, Suita, Japan; 5 Department of Immunoparasitology, Research Institute for Microbial Diseases, Osaka University, Suita, Japan; 6 Laboratory of Virus Control, Research Institute for Microbial Diseases, Osaka University, Suita, Japan; 7 Department of Molecular Virology, Research Institute for Microbial Diseases, Osaka University, Suita, Japan; 8 Osaka City University Graduate School of Medicine, Osaka, Japan; 9 Immunology Frontier Research Center, Osaka University, Suita, Japan; 10 Center for Infectious Disease Education and Research, Osaka University, Suita, Japan

**Keywords:** Senescence, Virology, Ageing

## Abstract

Reports of post-acute COVID-19 syndrome, in which the inflammatory response persists even after SARS-CoV-2 has disappeared, are increasing^1^, but the underlying mechanisms of post-acute COVID-19 syndrome remain unknown. Here, we show that SARS-CoV-2-infected cells trigger senescence-like cell-cycle arrest^2,3^ in neighboring uninfected cells in a paracrine manner via virus-induced cytokine production. In cultured human cells or bronchial organoids, these SASR-CoV-2 infection-induced senescent cells express high levels of a series of inflammatory factors known as senescence-associated secretory phenotypes (SASPs)^4^ in a sustained manner, even after SARS-CoV-2 is no longer detectable. We also show that the expression of the senescence marker *CDKN2A* (refs. ^5,6^) and various SASP factor^4^ genes is increased in the pulmonary cells of patients with severe post-acute COVID-19 syndrome. Furthermore, we find that mice exposed to a mouse-adapted strain of SARS-CoV-2 exhibit prolonged signs of cellular senescence and SASP in the lung at 14 days after infection when the virus was undetectable, which could be substantially reduced by the administration of senolytic drugs^7^. The sustained infection-induced paracrine senescence described here may be involved in the long-term inflammation caused by SARS-CoV-2 infection.

## Abstract (web summary)

This study shows that SARS-CoV-2 infection induces paracrine senescence in adjacent uninfected cells via virus-induced cytokine secretion. This resulted in a persistent inflammatory response associated with senescence even after SARS-CoV-2 disappearance.

## Main

Since its emergence as a global pandemic in December of 2019, over two hundred million people worldwide have been infected with severe acute respiratory syndrome coronavirus 2 (SARS-CoV-2)^8^, and a significant proportion of these people have post-acute coronavirus disease 2019 (COVID-19) syndrome, in which symptoms persist and/or sequelae occur after the virus has disappeared^1^. Therefore, a deeper understanding of the biological responses to SARS-CoV-2 infection, especially the persistent response, is also needed. Because COVID-19 tends to be more severe in older people, some important clues may exist in the relationship between SARS-CoV-2 infection and aging^9^. Although numerous changes in various biological responses are associated with aging^10^, the accumulation of senescent cells has recently attracted keen attention^2,11^. Cellular senescence is a state of irreversible cell-cycle arrest that can be induced by a variety of potentially oncogenic stimuli and thus is considered to serve as an important mechanism of tumor suppression^3,12^. However, unlike apoptotic cells, senescent cells do not die immediately and thereby accumulate throughout the body during the aging process^2,12^. Importantly, senescent cells are not merely nondividing, as they also develop a phenomenon called the SASP (ref.^13^) in which they secrete a variety of proinflammatory factors, such as inflammatory cytokines, chemokines, growth factors and extracellular matrix-degrading enzymes, into the extracellular fluid^14–16^. Thus, although the induction of cellular senescence acts primarily as a mechanism of tumour suppression, the excessive accumulation of senescent cells in vivo due to aging is thought to have adverse effects via SASP^11,13^. Indeed, the removal of senescent cells by genetic or pharmacological approaches reportedly delays the onset of aging-associated inflammatory diseases in aged mice^7,17^. These findings prompted us to examine the relationship between cellular senescence and SARS-CoV-2 infection.

**Fig. 1:**
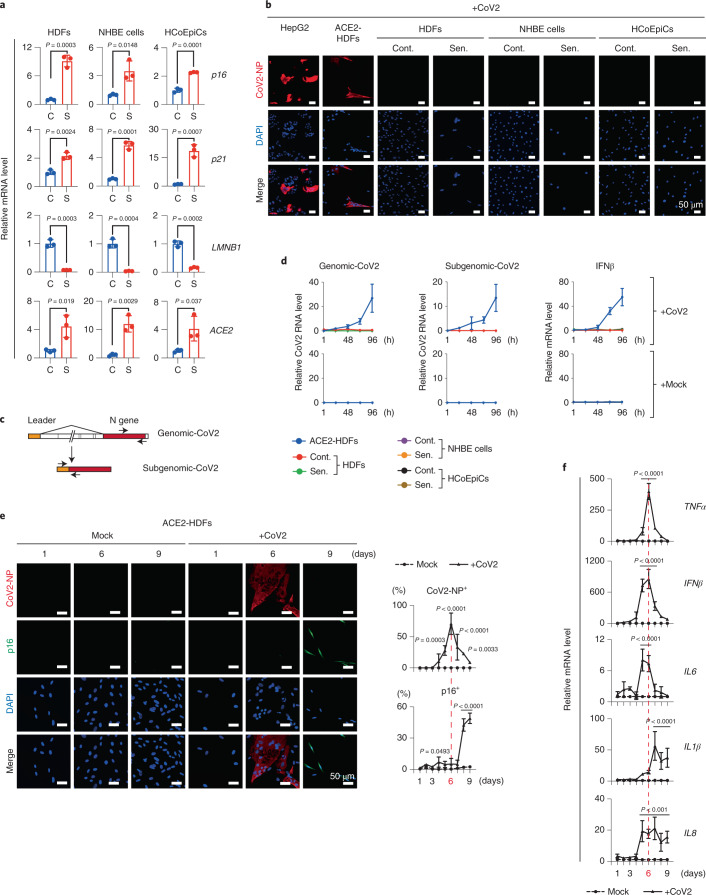
SARS-CoV-2 provoked a senescence-like phenotype. **a**–**d**, Early-passage HDFs (normal HDFs), normal human bronchial epithelial (NHBE) cells (normal human bronchial/tracheal epithelial cells), and HCoEpiCs (normal human colonic epithelial cells) were rendered senescence (S, red dots) by treatment with 250, 150 or 250 ng ml^−1^ doxorubicin, respectively, for 9 days. Vehicle-treated cells were used as a nonsenescence control (C, blue dots). The results of RT-qPCR analysis of senescence markers (*p16, p21* and *LMNB1*) and *ACE2* gene expression are shown (**a**). Senescent cells (Sen.) and nonsenescent control cells (Cont.) were infected with SARS-CoV-2 (+CoV2) at multiplicity of infection (m.o.i.) 1.0 for 4 days and fixed. HepG2 cells infected with SARS-CoV-2 at m.o.i. 1.0 for 24 h and ACE2-HDFs for 4 days were used as positive controls. Representative immunofluorescence images for SARS-CoV-2 N protein (CoV2-NP, red; DAPI, blue) are shown (**b**). Schematic diagram of genomic (genomic-CoV2) and subgenomic (subgenomic-CoV2) RNAs and the positions of the RT-qPCR primers used (**c**). Senescence (Sen.) and nonsenescence control (Cont.) cells infected with SARS-CoV-2 at m.o.i. of 1.0 were subjected to RT-qPCR analysis at 1, 24, 48, 72 and 96 h after infection, and the indicated genes were analyzed (**d**). **e**,**f**, ACE2-HDFs infected with (CoV2) or without (Mock) SARS-CoV-2 at m.o.i. of 1.0. were subjected to immunofluorescence staining for SARS-CoV-2 N protein (CoV2-NP (red)), p16 (green) and DAPI (blue) every day from days 1 to 9. Representative images of days 1, 6 and 9 are shown. The histograms indicate the percentages of cells expressing CoV2-NP (top) or p16^INK4a^ (bottom) (**e**). These cells were also subjected to RT-qPCR analysis for virus-induced cytokine and SASP factor gene expression (**f**). For all graphs, error bars indicate mean ± standard deviation (s.d.) of biological triplicate measurements. Statistical significance was determined with two-tailed unpaired Student’s *t* test (**a**) or two-way analysis of variance (ANOVA) followed by Sidak’s multiple comparison test in (**e**,**f**). Scale bars, 50 μm (**b**,**e**). Source data

**Extended Data Fig. 1:**
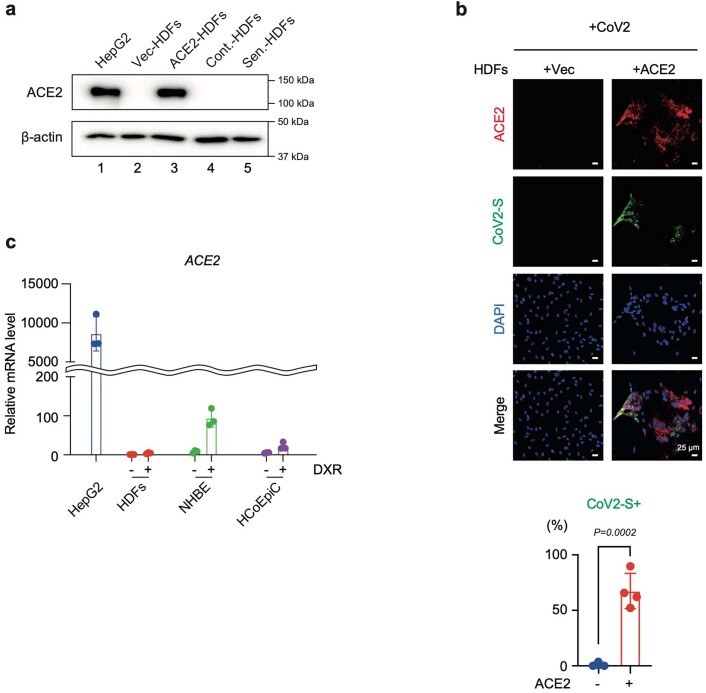
Ectopic expression of ACE2 allows HDFs to be infected with SARS-CoV-2. **a**, Western blotting analysis of ACE2 expression in early passage HDFs infected with a retrovirus encoding ACE2 (ACE2-HDFs) or an empty vector (Vec-HDFs), and doxorubicin-induced senescence HDFs (Sen.-HDFs) or control HDFs (Cont.-HDFs). HepG2 cells were used as a positive control. β-actin was used as a loading control. **b**, Vec-HDFs and ACE-HDFs were infected with SARS-CoV-2 at MOI of 1.0 for 6 days and were subjected to immunofluorescence analysis. Representative images for ACE2 [red], SARS-CoV-2 Spike protein (CoV2-S) [green] and DAPI [blue] were shown. The histogram shows the percentage of SARS-CoV-2 Spike protein expression in ACE2-expressing and non-expressing cells. For graphs, error bars indicate mean ± standard deviation (s.d.) of four biological replicates. **c**, Early passage HDFs, NHBE, and HCoEpiC were rendered senescence by treatment with doxorubicin (+DXR) for 9 days as described in Fig. 1. The levels of *ACE2* gene expression were examined by RT-qPCR using HepG2 cells as a positive control. For graphs, error bars indicate mean ± standard deviation (s.d.) of 3 biological replicates. Statistical significance was determined using Two-tailed unpaired t test in (b). Source data

**Extended Data Fig. 2:**
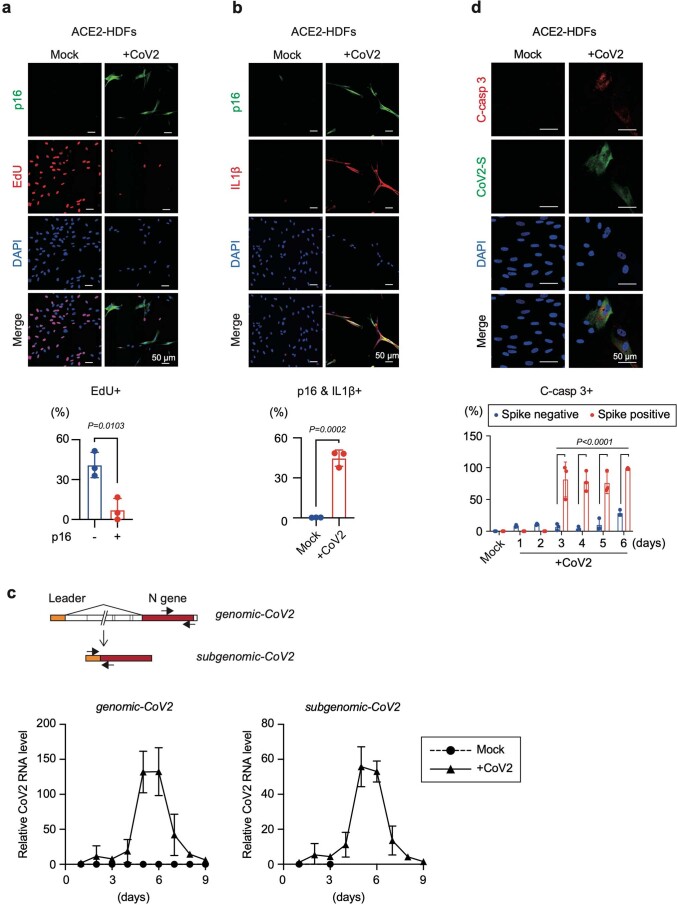
Signs of cellular senescence induced by SARS-CoV-2 infection. **a and b**, ACE2-HDFs were infected with (CoV2) or without (Mock) SARS-CoV-2 at MOI of 1.0 for 9 days. Immunofluorescence analysis of p16^INK4a^ expression [green], EdU incorporation [red], and DAPI [blue] (**a**), p16^INK4a^ expression [green], IL-1β [red], and DAPI [blue] (**b**) were shown. Histograms show the percentage of EdU positive in p16^INK4a^ negative (-) or positive (+) cells (**a**), double-positive cells for p16^INK4a^ expression, and IL-1β expression in Mock or CoV2 (**b**) on day 9 after SARS-CoV-2 infection. **c**, Schematic diagram of the genome (*genomic-CoV2*) and subgenomic (*subgenomic-CoV2*) RNAs and the positions of the RT-qPCR primers used. ACE2-HDFs infected with (CoV2) or without (Mock) SARS-CoV-2 at MOI of 1.0. were subjected RT-qPCR for *genomic-CoV2* and *subgenomic-CoV2* every day from day 1 to 9. **d**, SARS-CoV-2-infected ACE2-HDFs were subjected to immunofluorescence staining for cleaved caspase-3 (C-casp 3) [red], SARS-CoV-2 spike protein (CoV2-S) [green], and DAPI [blue] at indicated time point. A representative photograph at day 4 post-infection was shown (Scale bars, 50 μm). The histograms indicate the percentages of cleaved caspase-3 expressing cells with or without SARS-CoV-2 spike protein for every day from day 1 to 6 post-infection. For all graphs, error bars indicate mean ± standard deviation (s.d.) of biological triplicate. Statistical significance was determined using two-tailed unpaired Student’s t test in (a) and (b), and with two-way ANOVA followed by sidak’s multiple comparison test in (d). Source data

**Extended Data Fig. 3:**
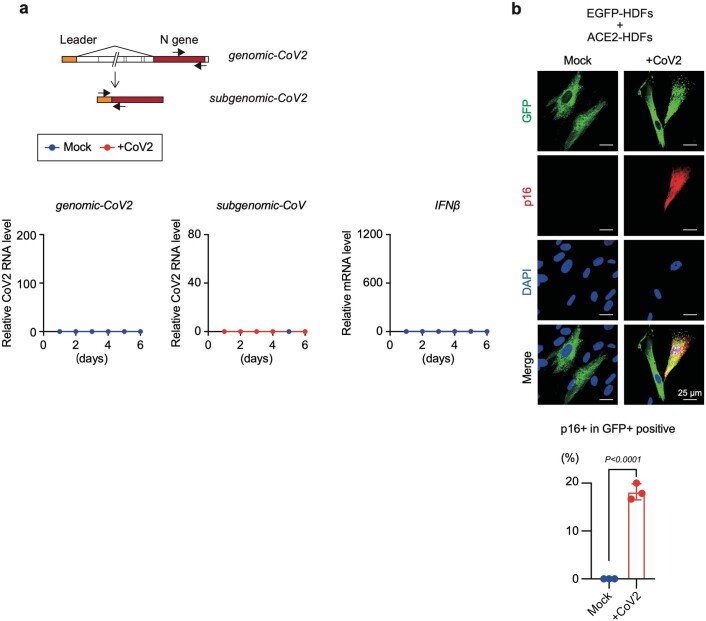
SARS-CoV-2-infected cells provoke senescence-like phenotype in neighboring cells. **a**, Schematic diagram of the genome (*genomic-CoV2*) and subgenomic (*subgenomic-CoV2*) RNAs and the positions of the RT-qPCR primers used. EGFP-HDFs administrated with (CoV2) or without (Mock) SARS-CoV-2 at MOI of 1.0. were subjected RT-qPCR for *genomic-CoV2*, *subgenomic-CoV2*, and *IFNβ* every day from day 1 to 6. **b**, EGFP-HDFs and ACE2-HDFs were cocultured in a 1:9 ratio, and SARS-CoV-2 was administered with (CoV2) or without (Mock) SARS-CoV-2 at an MOI of 1.0 for 9 days. Immunofluorescence images of EGFP [green], p16^INK4a^ [red], and DAPI [blue] were shown. The histogram indicated the percentages of p16^INK4a^ positive cells in EGFP positive cells. For all graphs, error bars indicate mean ± standard deviation (s.d.) of biological triplicate. Statistical significance was determined two-tailed unpaired Student’s t test in (b). Source data

Toward this purpose, we first set up a system to infect senescent cells with SARS-CoV-2 using primary normal human lung diploid fibroblasts (HDFs), which are commonly used in senescence studies^12^. Interestingly, the expression level of the *ACE2* gene, which is required for SARS-CoV-2 to enter the cells, was increased several-fold upon the induction of cellular senescence in HDFs, as well as in other cultured primary normal human cells (Fig. 1a). This is fairly consistent with the previous observation that the levels of the *ACE2* gene expression are slightly increased in lung tissues with age^18^. However, judging from the expression of the proteins encoded by SARS-CoV-2 and the detection of SARS-CoV-2 subgenomic RNA by Reverse transcription-quantitative polymerase chain reaction (RT-qPCR), we were unable to detect SARS-CoV-2 infection in these primary normal human cells, regardless of cellular senescence induction (Fig. 1b–d). Moreover, when ACE2 was ectopically expressed in HDFs (ACE2-HDFs) to a level comparable to that in HepG2 cells, a human cancer cell line that can be infected with SARS-CoV-2, the HDFs became infectable with SARS-CoV-2 (Fig. 1b–d and Extended Data Fig. 1a,b). Thus, although ACE2 expression tends to increase to a certain level in senescent cells, it is not high enough for SARS-CoV-2 infection, at least in these cultured primary normal human cells (Fig. 1a–d and Extended Data Fig. 1a,c). However, during this experiment, we unexpectedly noticed that signs of cellular senescence, such as cell-cycle arrest (absence of EdU incorporation)^3^, induction of expression of p16^INK4a^ (ref. ^6^; one of the proteins encoded by the *CDKN2a* gene locus^5^) and interleukin-1β (IL-1β) (SASP factor) expression^14^ were observed on day 9 after SARS-CoV-2 administration (Extended Data Fig. 2a,b). This is somewhat consistent with previous reports that certain viral infections can directly induced senescence-like features in cultured cells^19^. Curiously, most of these senescence-like cells no longer expressed the protein encoded by SARS-CoV-2, and the SARS-CoV-2 subgenomic RNA was hardly detectable by qPCR on day 9 after SARS-CoV-2 administration to ACE2-HDFs (Fig. 1e, and Extended Data Fig. 2c), indicating that SARS-CoV-2 either decreased below the detection limit after inducing the senescence-like phenotype or indirectly prompted this phenotype in uninfected cells. To clarify this point, HDFs ectopically expressing EGFP (EGFP-HDFs), which cannot be infected with SARS-CoV-2 (Extended Data Fig. 3a), were cocultured with the HDFs ectopically expressing ACE2 (described above, ACE2-HDFs) and administered SARS-CoV-2. Intriguingly, a significant proportion of EGFP-HDFs were positive for p16^INK4a^ (Extended Data Fig. 3b), suggesting that SARS-CoV-2 indirectly induces the senescence-like phenotype.

**Fig. 2:**
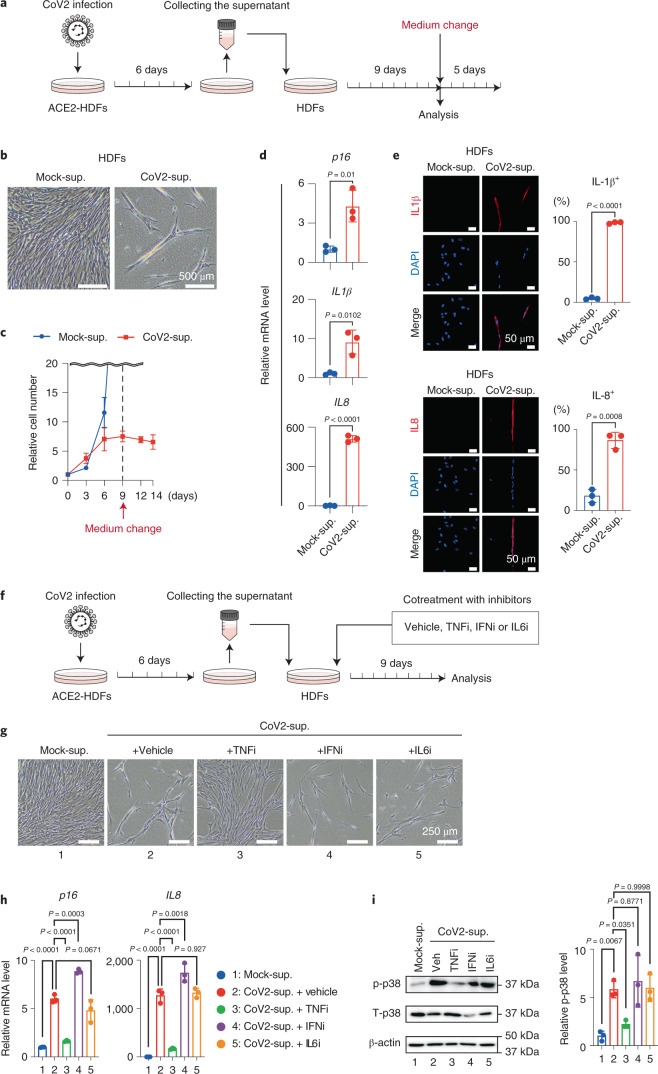
SARS-CoV-2-infected cells induced paracrine senescence via TNF. **a**–**e**, Early-passage HDFs were cultured for 9 days with culture supernatant of ACE2-HDFs infected with SARS-CoV-2 for 6 days and then analyzed or cultured in plain tissue culture medium for another 5 days. Experimental design (**a**). Representative photographs of HDFs on day 9 after incubation with culture supernatant of ACE2-HDFs infected with (CoV2-sup.) or without (Mock-sup.) SARS-CoV-2 (**b**). Relative cell number counted throughout the experiments (**c**), gene expression analysis on day 9 by RT-qPCR (**d**) and immunofluorescence staining (**e**) for indicated genes or proteins on day 9. **f**–**i**, Early-passage HDFs were cultured for 9 days with culture supernatant of ACE2-HDFs infected with SARS-CoV-2 for 6 days in the presence or absence of indicated cytokine-inhibitors (TNF-α inhibitor (TNFi), 500 μg ml^−1^ etanercept; IL-6 inhibitor (IL6i), 50 μg ml^−1^ tocilizumab; and type I IFN neutralizing antibody (IFNi), 1:200). Experimental design (**f**). Representative photographs of the cells on day 9 (**g**). RT-qPCR analysis of day 9 expression of the genes shown at the top of **h**. Immunoblotting of phospho-p38 and total p38, (left) and relative density of phospho-p38 normalized by total p38 and β-actin using ImageJ (right) (**i**). Veh, vehicle. β-Actin was used as a loading control. For all graphs, error bars indicate mean ± s.d. of biological triplicate measurements. Statistical significance was determined with a two-tailed unpaired Student’s *t* test in (**d**,**e**) and one-way ANOVA followed by Dunnett’s multiple comparison test (**h**,**i**). Scale bars, 50 μm (**e**), 250 μm (**g**) and 500 μm (**b**). Source data

**Extended Data Fig. 4:**
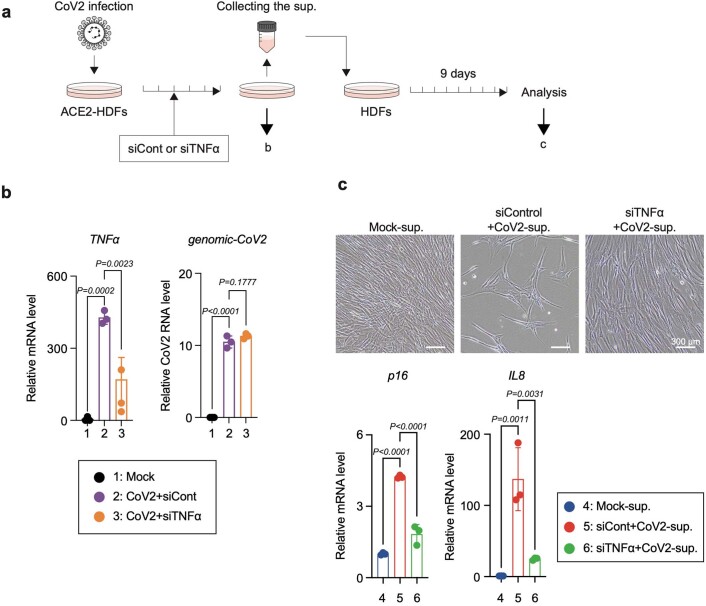
Depletion of TNF-α from SARS-CoV-2-infected cells by siRNA reduces senescence-inducing activity. **a**, Early passage HDFs were cultured for 9 days with culture supernatant of ACE2-HDFs infected with SARS-CoV-2 for 6 days in the presence of indicated siRNA. **b**, RT-qPCR analysis of *TNFα* and *genomic RNA* of SARS-CoV-2 in ACE2-HDFs infected with SARS-CoV-2 with or without siRNA-mediated depletion of indicated genes. **c**, Representative photographs (upper panel) and RT-qPCR analysis (lower panel) of HDFs incubated with culture supernatant of SARS-CoV-2-infected ACE2-HDFs for 9 days (upper panels). For all graphs, error bars indicate mean ± standard deviation (s.d.) of biological triplicate. Statistical significance was determined with one-way ANOVA followed by Dunnett’s multiple comparison test in (b) and (c). Source data

To determine how SARS-CoV-2 indirectly induces the senescence-like phenotype, we compared the time course of SARS-CoV-2 infection and senescence-like phenotype induction in ACE2-HDFs. Notably, SARS-CoV-2-infected cells became detectable as early as day 4 after SARS-CoV-2 administration and reached a peak on day 6, but the signs of cellular senescence appeared later, reaching a peak on day 9 when most of the SARS-CoV-2-infected cells had died (Fig. 1e, Extended Data Fig. 2c and d). These results are consistent with the observation that the expression levels of virus-induced cytokines, such as *IFNβ, IL6* and *TNFα*, peak on day 6 after infection with SARS-CoV-2 and then decrease markedly, whereas those of other cytokines belonging to the SASP factor, such as *IL1β* and *IL8*, persist even after SARS-CoV-2 is no longer detected (Fig. 1f). Some of these virus-induced cytokines reportedly promote cellular senescence, depending on the biological context^15,16^. Therefore, we next tested whether SARS-CoV-2 infection causes the senescence-like phenotype indirectly through these virus-induced cytokines. The incubation of HDFs incapable of SARS-CoV-2 infection with culture supernatants of ACE2-HDFs 6 days after SARS-CoV-2 infection did indeed elicit senescence-like phenotypes, such as irreversible cell-cycle arrest and expression of *p16*^*INK4a*^ (ref. ^6^; one of the genes encoded by *CDKN2A* gene locus^5^) and SASP (Fig. 2a to e). Notably, this effect was greatly attenuated in the presence of the anti-tumor necrosis factor (anti-TNF) agent, but not the anti-type I interferon (IFN) agent or anti-IL-6 agent (Fig. 2f–h). Furthermore, similar results were obtained by depleting TNF-α in SARS-CoV-2-infected cells with short interfering RNA before collecting the culture supernatant (Extended Data Fig. 4). These results suggest that TNF-α plays an important role, at least partially, in the induction of the senescence-like phenotype of HDFs by SARS-CoV-2.

**Fig. 3:**
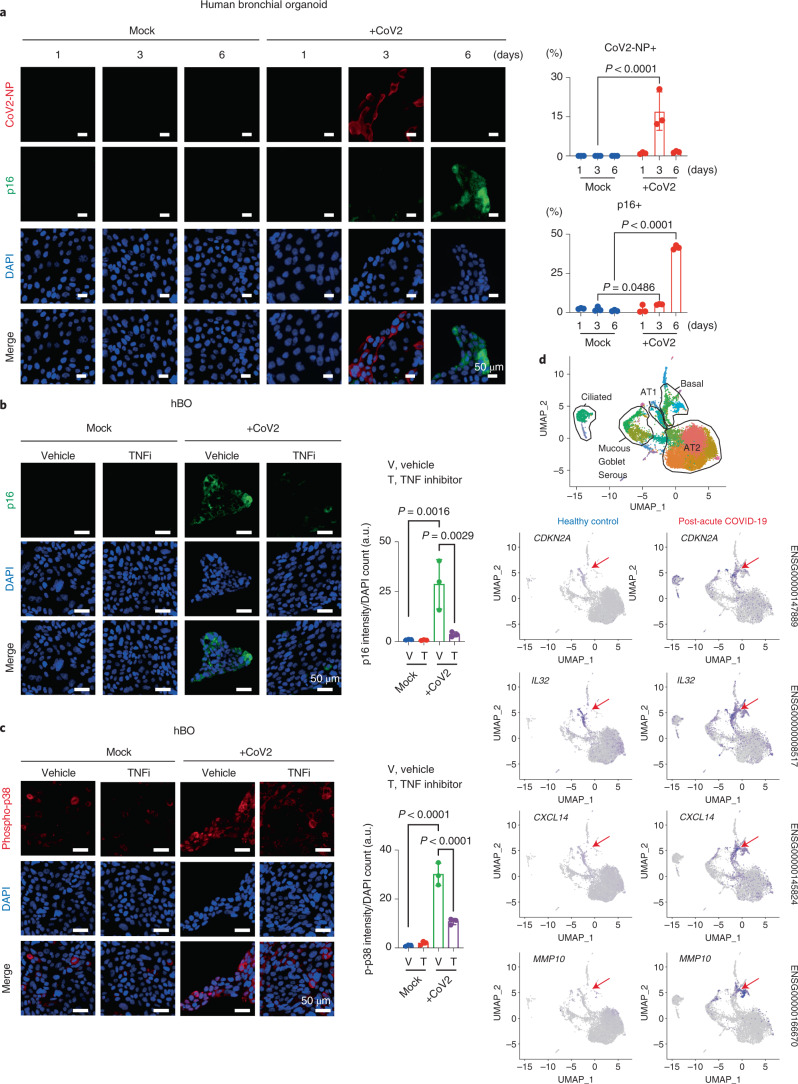
SARS-CoV-2 provoked a senescence-like phenotype in hBOs and patients with post-acute COVID-19. **a**, hBOs infected with SARS-CoV-2 at m.o.i. 0.1 were cultured for 6 days and then subjected to immunofluorescence staining for SARS-CoV-2 N protein (CoV2-NP (red), p16^INK4a^ (green) and DAPI (blue)). Representative images of days 1, 3 and 6 are shown. The histograms indicate the percentages of cells expressing COV2-NP (top) or p16^INK4a^ (bottom). **b**,**c**, hBOs infected with SARS-CoV-2 at m.o.i. 0.1 were treated with a TNF-α inhibitor (TNFi) (500 μg ml^−1^ etanercept) from day 3 after infection. Immunofluorescence staining for p16^INK4a^ (green) and DAPI (blue) (**b**) or phospho-p38 (red) and DAPI [blue] (**c**) is shown. The histograms indicate the intensity of p16^INK4a^ signals or phospho-p38 signals normalized by DAPI count. **d**, Single-cell RNA transcriptomic analysis of lung tissue from patients with severe COVID-19 (ref. ^28^). Epithelial cells population of uniform manifold approximation and projection (UMAP) plots were divided into healthy controls (25 and 52 years old) and patients with COVID-19 (28, 54 and 57 years old), and the distribution of cells expressing senescent cell marker (*CDKN2A*) and SASP factor genes is shown. Arrowheads indicate a basal cell population. For all graphs, error bars indicate mean ± s.d. of biological triplicate measurements. Scale bars, 50 μm (**a–c**). Statistical significance was determined by two-way ANOVA followed by Sidak’s multiple comparison test (**a**) or one-way ANOVA followed by Dunnett’s multiple comparison test (**b**,**c**). a.u., arbitrary units. Source data

**Extended Data Fig. 5:**
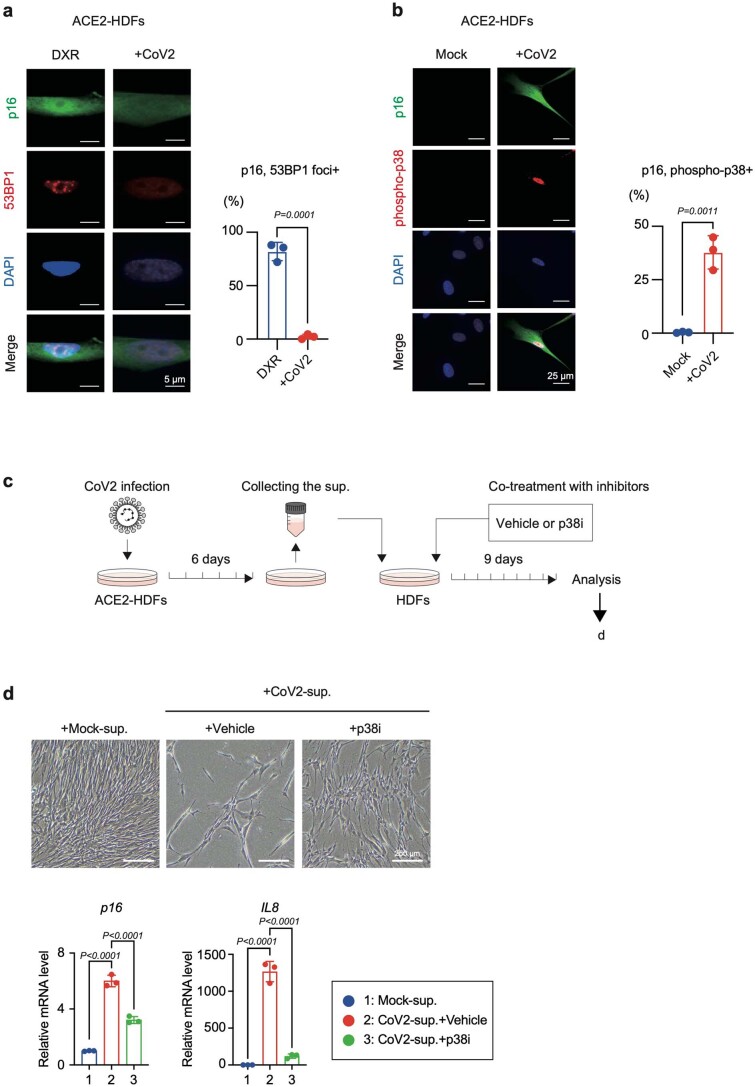
SARS-CoV-2 infection promotes phosphorylation of p38 in human fibroblasts. **a**, ACE2-HDFs were rendered senescence by treatment with 250 ng/ml of doxorubicin (DXR) or by infection with SARS-CoV-2 MOI of 1.0 for 9 days. Immunofluorescence images of p16^INK4a^ [green], 53BP1 [red], and DAPI [blue] were shown. The histogram indicated the percentages of 53BP1 foci positive cells in p16^INK4a^ positive cells. DXR treated ACE2-HDFs were positive control of p16^INK4a^ and 53BP1 foci. **b**, Immunofluorescence images of p16^INK4a^ [green], phosphorylated-p38 [red], and DAPI [blue] were shown. The histogram indicated the percentages of phosphorylated-p38 positive cells in p16^INK4a^ positive cells. **c-d**. Early passage HDFs were cultured with a conditioned medium of ACE2-HDFs infected with SARS-CoV-2 for 9 days in the presence or absence of 10 μM of p38 inhibitor, SB203580 (p38i). Experimental design (**c**), representative photographs of the cells (upper panel), and RT-qPCR analysis for indicated genes (**d**) on day 9 were shown. For all graphs, error bars indicate mean ± standard deviation (s.d.) of biological triplicate. Statistical significance was determined with two-tailed unpaired Student’s t test in (a) and (b), and with one-way ANOVA followed by Dunnett’s multiple comparison test in (d). Source data

**Extended Data Fig. 6:**
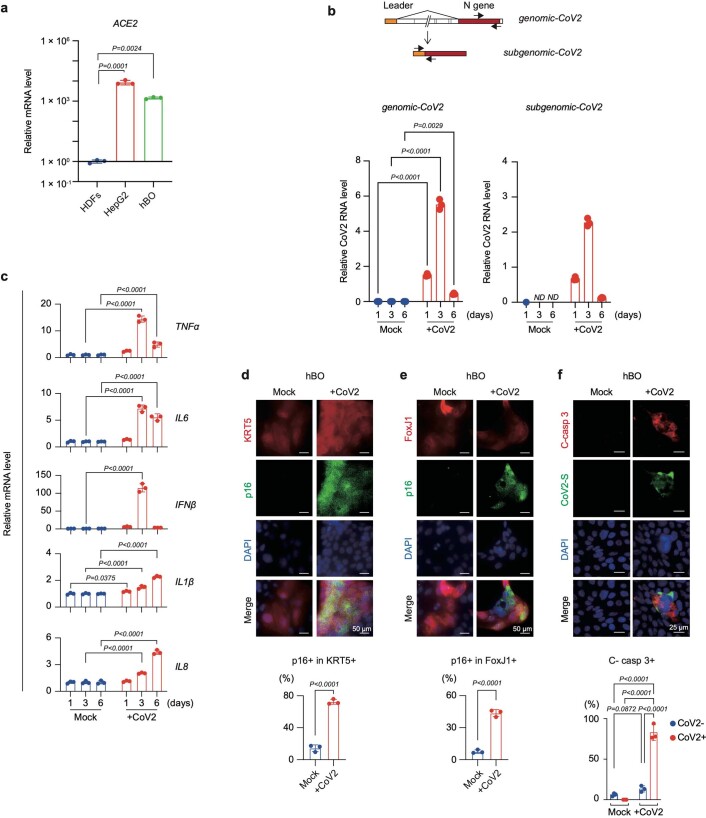
SARS-CoV-2 provokes senescence-like phenotype in hBOs. **a**, RT-qPCR analysis of *ACE2* gene expression in HDFs, HepG2, and human bronchial organoid (hBO). **b**, Schematic diagram of the genome (*genomic-CoV2*) and subgenomic (*subgenomic-CoV2*) RNAs and the positions of the RT-qPCR primers used (upper panel). hBOs were infected with SARS-CoV-2 at MOI of 0.1 for the indicated periods (lower panel). These cells were analyzed by RT-qPCR to determine the genomic and subgenomic SARS-CoV-2 RNAs (lower panel), gene expression of virus-induced cytokines and SASP factors (**c**), or by immunofluorescence analysis of KRT5 (basal cell marker) expression [red], p16^INK4a^ expression [green], and DAPI [blue] (**d**), FoxJ1 (ciliated cell marker) expression [red], p16INK4a expression [green], and DAPI [blue] (**e**) or cleaved caspase-3 (C-casp 3) [red], SARS-CoV-2 spike protein (CoV2-S) [green], and DAPI [blue] (**f**) in Mock or SARS-CoV-2-infected cells (CoV2) on day 6 after SARS-CoV-2 infection. (Scale bars, 50 μm). The histograms indicate the percentages of p16^INK4a^ expressing cells in KRT5 or FoxJ1 positive cell, and cleaved caspase-3 positive cell percentages in SARS-CoV-2 spike protein negative (CoV2-) or positive (CoV2 + ) cells. For all graphs, error bars indicate mean ± standard deviation (s.d.) of biological triplicate. Statistical significance was determined with two-tailed unpaired Student’s t test in (a), (d) and (e), and with two-way ANOVA followed by sidak’s multiple comparison test in (b), (c), and (f). Source data

**Extended Data Fig. 7:**
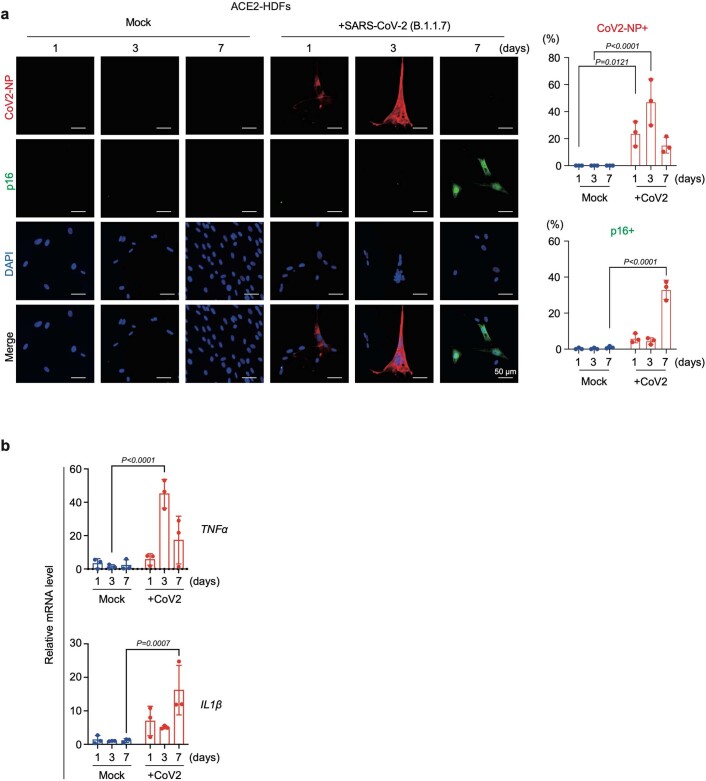
SARS-CoV-2 (B.1.1.7) variant provokes senescence-like phenotype. **a**,**b**, ACE2-HDFs infected with (CoV2) or without (Mock) SARS-CoV-2 (B.1.1.7) variant at MOI of 1.0. were subjected to immunofluorescence staining for SARS-CoV-2 N protein (CoV2-NP [red]), p16^INK4a^ [green], and DAPI [blue] daily from day 1 to day 9. Representative images of day 1, day 3, and day 7 were shown (**a**). The histograms indicate the percentages of COV2-NP (upper) or p16^INK4a^ (lower) expressing cells. These cells were also subjected to RT-qPCR for virus induce cytokines and SASP factor (**b**). For all graphs, error bars indicate mean ± standard deviation (s.d.) of biological triplicate. Statistical significance was determined with two-way ANOVA followed by sidak’s multiple comparison test in (**a**) and (**b**). Source data

Although 53BP1 foci formation, a sign of DNA damage response^3^, was not observed, p38 MAP kinase, a downstream signaling mediator of TNF-α^20^, is highly phosphorylated in SARS-CoV-2-induced senescence-like HDFs (Extended Data Fig. 5a,b). Moreover, levels of p38 phosphorylation were reduced by treatment with the anti-TNF agent, but not anti-type I-IFN agent or anti-IL-6 agent (Fig. 2i). Consistent with this observation, the induction of the senescence-like phenotype by culture supernatants from ACE2-HDFs infected with SARS-CoV-2 was partially attenuated by treatment with a p38 inhibitor (Extended Data Fig. 5c,d). These results, in conjunction with previous observations that TNF-α causes phosphorylation and activation of p38 and activated p38 induces *p16*^*INK4a*^ expression and SASP in a DNA damage response-independent manner^20–24^, strongly suggest that SARS-CoV-2 provokes a senescence-like phenotype at least partly through TNF-α/p38 pathway activation. However, because these results were obtained using HDFs ectopically expressing ACE2, we sought evidence that a similar phenotype can be induced in normal human cells without ectopic ACE2 expression. To this end, we used human bronchial organoids (hBOs), which highly express endogenous ACE2 (ref. ^25^) (Extended Data Fig. 6a). Notably, the administration of SARS-CoV-2 to hBOs induced signs of cellular senescence after SARS-CoV-2 became undetectable, as in the case of ACE2-HDFs (Fig. 3a and Extended Data Fig. 6b–e). Furthermore, a substantial proportion of SARS-CoV-2-infected hBOs expressed activated caspase-3 (Extended Data Fig. 6f), indicating that SARS-CoV-2-infected cells are more susceptible to death, as seen in ACE2-HDFs (Extended Data Fig. 2d). This may explain why most of the senescent cells that emerge after SARS-CoV-2 becomes undetectable are SARS-CoV-2 uninfected. Furthermore, treatment with the anti-TNF agent substantially suppressed the induction of p16^INK4a^ expression and phosphorylation of p38 in SARS-CoV-2-infected hBOs (Fig. 3b,c), ruling out the possibility that the above results simply reflect an artifact of ectopic ACE2 overexpression. Interestingly, people who have developed post-acute sequelae of SARS-CoV-2 infection reportedly have higher levels of cytokines such as TNF-α in the early stages of recovery^26^, consistent with our experimental data. Together, these results indicate that, at least in certain biological contexts, SARS-CoV-2 provokes paracrine senescence via cytokines secreted by infected cells. It should also be noted that the more virulent SARS-CoV-2 variant (B.1.1.7), first observed in the United Kingdom^27^, also induced a senescence-like phenotype in ACE2-HDFs with slightly faster kinetics than the original SARS-CoV-2 (Extended Data Fig. 7), suggesting that the induction of the senescence-like phenotype is common in normal human cells infected with SARS-CoV-2.

Considering that SASP sustains inflammatory responses^4,13–16^ and that the persistence of inflammation is likely to be one of the causes of post-acute COVID-19 syndrome^1^, we next explored the possibility that the senescence-like phenotype contributed to the sustained inflammatory response in post-acute COVID-19 syndrome. To this end, we reanalyzed data from a recently published single-cell transcriptomic analysis of lung tissue from patients with severe COVID-19 (ref. ^28^). In that study, SARS-CoV-2 was no longer detected in the patients with prolonged COVID-19 disease, but the pathology showed extensive evidence of damage and fibrosis resembling end-stage pulmonary fibrosis^28^. Notably, the expression levels of *p16*^*INK4a*^ (*CDKN2A*) and several SASP factor genes, such as *IL32, CXCL14* and *MMP10*^29–31^, were increased in the lung cells of patients with severe COVID-19 as compared with those of healthy individuals, especially in basal cells and, to a lesser extent, in ciliated cells (Fig. 3d and Supplementary Fig. 1). This is consistent with the observation that a significant proportion of basal cells and a slightly smaller proportion of ciliated cells express p16^INK4a^ in SARS-CoV-2-infected hBOs after SARS-CoV-2 is no longer detectable (Fig. 3a and Extended Data Fig. 6b,d,e). Thus, it is tempting to speculate that the senescence-like phenotype induced by SARS-CoV-2 infection may be involved in the development of post-acute COVID-19 syndrome.

**Fig. 4:**
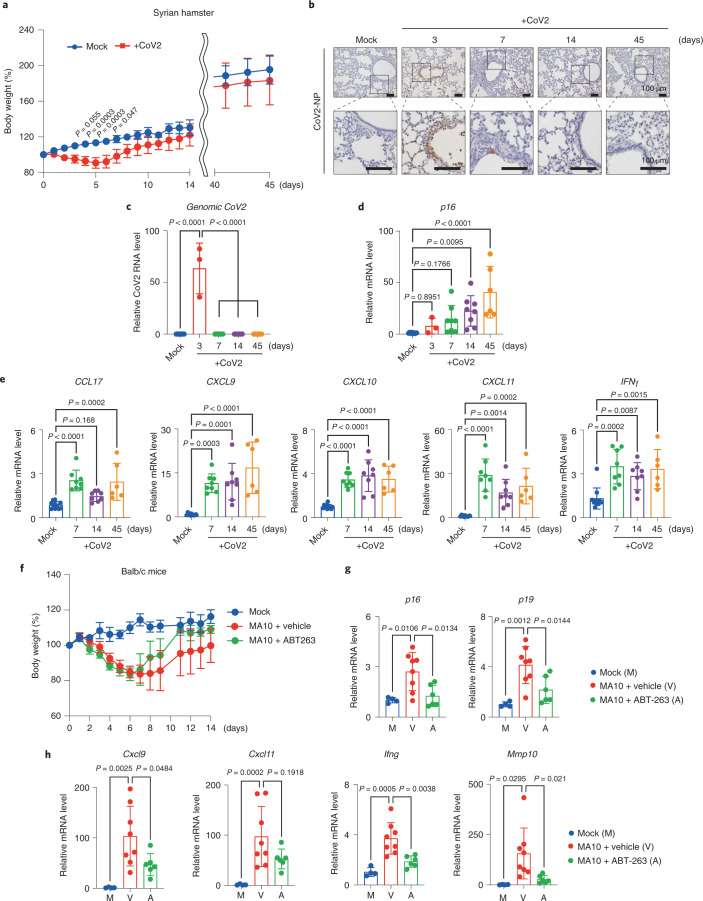
SARS-CoV-2 infection causes a senescence-associated inflammatory response. **a**, Syrian hamsters were intranasally inoculated with SARS-CoV-2 (B.1.1.7) 5.6 × 10^5^ plaque-forming units (p.f.u.; in 80 μl) or medium (mock). The body weight of mock-infected or SARS-CoV-2 (CoV2)-infected hamsters was monitored until day 14 (mock, *n* = 6; CoV-2, *n* = 8) and day 45 (mock, *n* = 3; CoV2, *n* = 6). Data are presented as the mean percentages of the starting weight (±s.d.). **b**–**e**, Syrian hamsters were infected with SARS-CoV-2 (B.1.1.7) (CoV2) or mock and euthanized on days 3 (*n* = 3), 7 (*n* = 8), 14 (*n* = 8) and 45 *(n* = 6) after infection. Immunohistochemistry images of SARS-CoV-2 N protein (CoV2-NP) staining are shown (scale bars, 100 μm) (**b**). RT-qPCR analysis of SARS-CoV-2 virus genomic RNA by RT-qPCR (**c**), *p16*^*INK4a*^ (**d**) and SASP factor expression (**e**). **f**–**h**, BALB/c mice were intranasally inoculated with MA10, 1.0 × 10^4^ median tissue culture infectious dose (TCID_50_) (in 50 μl) and treated with ABT-263 (*n* = 6), vehicle (*n* = 8) or mock infection (*n* = 4). The body weight was monitored until day 14 (**f**). The level of gene expression of senescence markers (**g**) and SASP factors (**h**) was analyzed by RT-qPCR. For all graphs, error bars indicate mean ± s.d. The y-axis shows the relative amount of RNA when the amount of RNA in Mock is set to 1 (**c**–**e**,**g**,**h**). Statistical significance was determined by two-way ANOVA followed by Sidak’s multiple comparison test (**a**) or one-way ANOVA followed by Dunnett’s multiple comparison test (**c**–**e**,**g**,**h**). Source data

**Extended Data Fig. 8:**
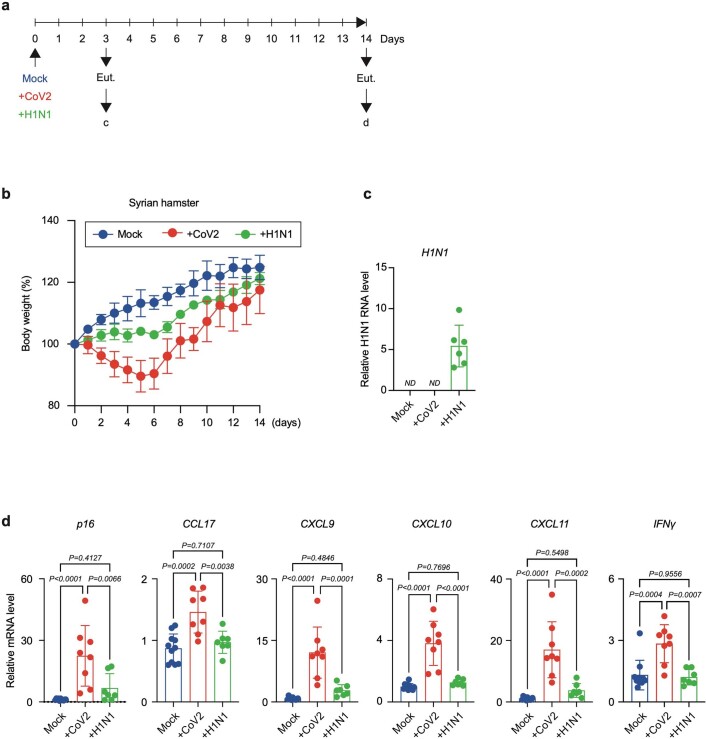
The mild seasonal influenza A (H1N1) did not cause a senescence-like phenotype. **a**–**d**. Syrian hamsters were intranasally inoculated with SARS-CoV-2 (B.1.1.7) 5.6 × 10^5^ PFU (in 80 μL), medium (mock) as described in Fig. 4, or influenza A (H1N1) pdm09 5.6 × 10^4 PFU (80 μL). Timeline of experiment (**a**). The body weight were monitored until day 14 (mock; n = 6, CoV2; n = 8, H1N1; n = 7) (**b**). Syrian hamsters were infected with SARS-CoV-2 (CoV2) (n = 3), mock (n = 3), or H1N1 (n = 6) and euthanized on day 3 post-infection. The H1N1 infection was monitored by RT-qPCR (**c**). The hamsters infected with these viruses were euthanized on day 14 post-infection (mock; n = 11, CoV2; n = 8, H1N1; n = 7), and were subjected to RT-qPCR analysis for the expression of senescence marker (*p16*^*INK4a*^) and SASP factors gene (**d**). The data for mock and SARS-CoV-2-infected hamsters are the same as those used in Fig. 4a–e. For all graphs, error bars indicate mean ± standard deviation (s.d.). Statistical significance was determined with one-way ANOVA followed by Tukey multiple comparison tests in (d). Source data

**Extended Data Fig. 9:**
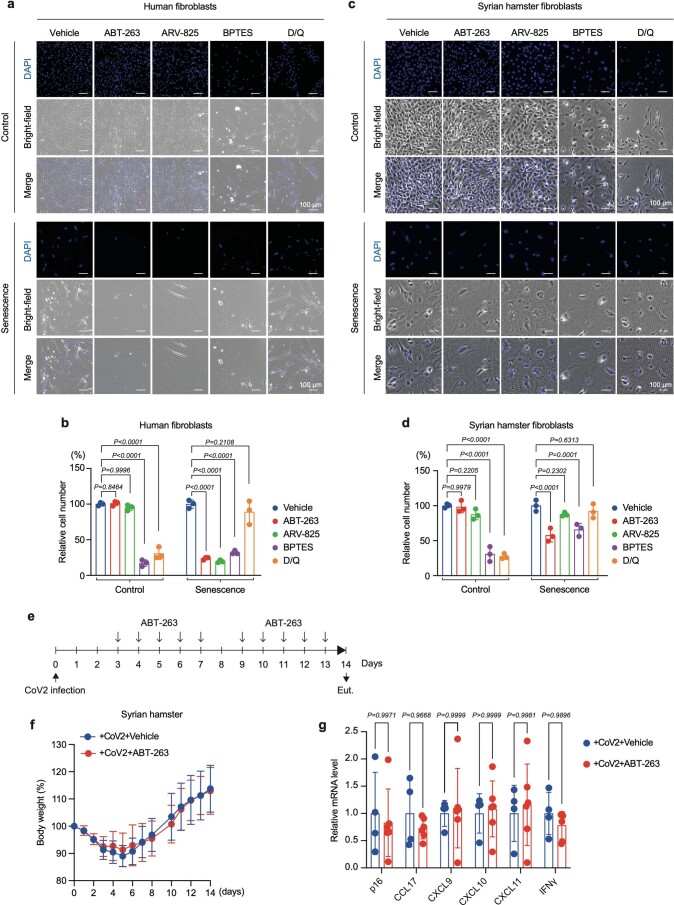
Existing senolytic drugs were ineffective in Syrian hamsters both in vitro and in vivo. **a**–**d**, The early passage human diploid fibroblasts (HDFs) and Syrian hamster fibroblasts (Control) were rendered senescence by treatment with doxorubicin (Senescence) for 9 days. Then, control and senescence human/Syrian hamster fibroblasts were cultured with indicated senolytic drugs (ABT-263; 1 μM, ARV-825; 25 nM, BPTES; 10 μM, dasatinib; 2 μM /quercetin; 20 μM (D/Q)) for 72 hours. Representative photographs of 3 biological replicates were shown (**a**,**c**). The relative cell number of 3 biological replicates (vehicle-treatment cells were calculated as 100%) was shown (**b and d**). **e**–**g**, Syrian hamsters were intranasally inoculated with SARS-CoV-2 (B.1.1.7) 5.6 × 10^5^ PFU (in 80 μL), and treated with ABT-263 (100 mg/kg, i.p.) or vehicle. The timeline of the experiment was shown (**e**). The body weight were monitored until day 14 (CoV2+vehicle; n = 4, CoV2+ABT-263; n = 6) (**f**). The senescence-related gene expression at day 14 post-infection by RT-qPCR analysis (CoV2+vehicle; n = 4, CoV2+ABT-263; n = 6) was shown (**g**). For all graphs, error bars indicate mean ± standard deviation (s.d.).Statistical significance was determined with two-way ANOVA followed by sidak’s multiple comparison test in (b), (d), and (e). Source data

**Extended Data Fig. 10:**
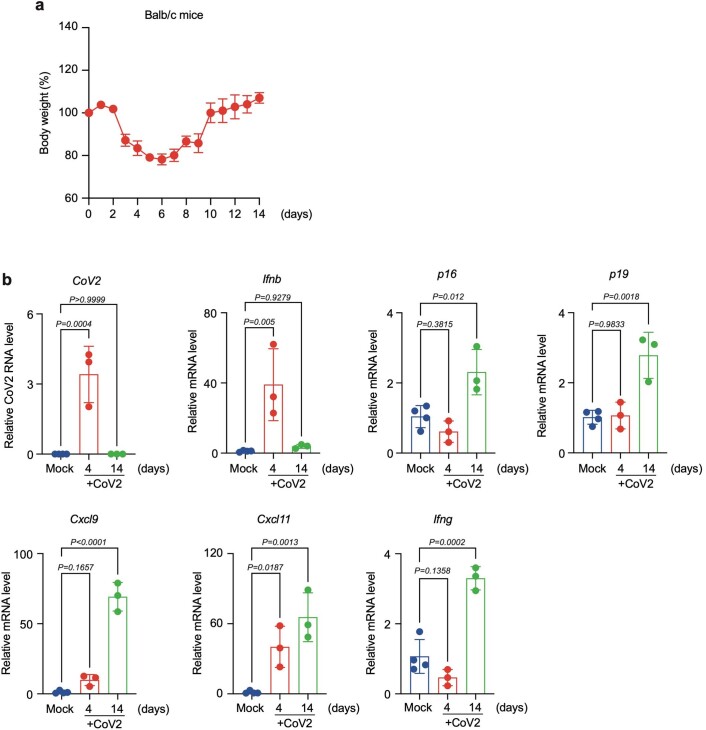
In MA10-infected Balb/c mice, senescence-like phenotypes appeared in inverse proportion to the decrease in the virus. **a**,**b**, Balb/c mice were intranasally inoculated with MA10, 1.0 × 10^4^ TCID50 (in 50 μL). The body weight was monitored until day 14 (n = 3) (**a**). MA10 infected balb/c mice were euthanized on day 4 (n = 3) and day 14 (n = 3) post-infection, and RT-qPCR analysis was performed to examine the indicated gene expression. The data for mock and SARS-CoV-2-infected hamsters are the same as those used in Fig. 4a–e. For all graphs, error bars indicate mean ± standard deviation (s.d.). Statistical significance was determined with one-way ANOVA followed by Dunnett’s multiple comparison test in (b). Source data

To further explore this possibility, we next examined whether SARS-CoV-2 infection induced *p16*^*INK4a*^ expression in vivo using Syrian hamsters. Unlike mice, hamsters can be infected with SARS-CoV-2 (ref. ^32^) (Fig. 4a–c), and the expression level of *p16*^*INK4a*^ was significantly increased from day 7 after infection in the lung, when SARS-CoV-2 became hardly detectable in hamsters (Fig. 4b–d). This is consistent with the observation that a substantial proportion of SARS-CoV-2-infected cells have positive TdT-mediated dUTP nick end labelling (TUNEL) staining, a marker of apoptosis, at day 5 after infection (Supplementary Fig. 2). Moreover, the expression levels of *p16*^*INK4a*^ and SASP factors remained high even at days 14 and 45, when SARS-CoV-2 was almost undetectable (Fig. 4c–e), indicating that the senescence-associated inflammatory response may persist to some extent even after SARS-CoV-2 decreased below the detection limit in Syrian hamsters. Notably, this phenomenon was not observed when Syrian hamsters were infected with the influenza A (H1N1) virus, a respiratory virus that does not cause long-term symptoms after recovery^1,33^ (Extended Data Fig. 8), suggesting that this phenomenon is likely to be unique to SARS-CoV-2 infection. To further prove that SARS-CoV-2 provokes and sustains a senescence-associated inflammatory response, we attempted to eliminate senescent cells in Syrian hamsters by using senolytic drugs that can selectively kill senescent cells. Among the reported senolytic drugs^7^ we tested, ABT-263 (ref. ^34^) and ARV-825 (ref. ^35^) specifically reduced the number of senescent human fibroblasts at the reported optimal concentrations (Extended Data Fig. 9a,b). However, in hamster fibroblasts, only ABT-263 showed slight senolytic activity (Extended Data Fig. 9c,d). Moreover, the administration of ABT-263 failed to decrease the expression levels of *p16*^*INK4a*^ and SASP factors in Syrian hamsters infected with SARS-CoV-2 (Extended Data Fig. 9e–g), suggesting that some differences may exist in the pathways that regulate the survival of senescent cells between humans and hamsters. To circumvent this problem, we used mice that are known to be sensitive to senolytic drugs^7^. To this end, we used a mouse-adapted strain of SARS-CoV-2 (MA10) (ref. ^36^), which shows dose- and age-related increases in pathogenesis in standard laboratory mice and recapitulates key features of COVID-19 in humans^36^. Similar to the Syrian hamsters, signs of cellular senescence, including expression of *p16*^*INK4a*^ and *p19*^*ARF*^ (critical inducers of cellular senescence encoded by the *CDKN2A* gene) and SASP factor genes, were increased at day 14 after infection in the lung, when SARS-CoV-2 became hardly detectable in BALB/c mice (Extended Data Fig. 10). Intriguingly, the administration of ABT-263 substantially decreased the expression levels of these senescence-associated genes in BALB/c mice infected with MA10 (Fig. 4f–h), further supporting the idea that SARS-CoV-2 infection provokes and sustains senescence-associated inflammatory phenotypes even after SARS-CoV-2 is no longer detectable in vivo.

During the preparation of this article, Camell et al. reported that the elimination of senescent cells, termed senolysis, reduced mortality in aged mice infected with mouse hepatitis virus (MHV), a virus in the same family as SARS-CoV-1 and 2^37^. However, the mechanisms involved in viral entry into cells and the pathogenesis are very different between MHV and SARS-CoV-2 (ref. ^38^), and thus, the extent to which the data obtained from MHV can be applied to SARS-CoV-2 remains unclear. In addition, Lee et al. have recently reported that SARS-CoV-2 rapidly causes senescent phenotypes in infected cells within 5 days of infection in both cultured human cells ectopically expressing ACE2 and Syrian hamsters^39^. They were also able to eliminate senescent cells by administering senolytic drugs such as ABT-263 or dasatinib plus quercetin (D + Q) to SARS-CoV-2-infected hamsters using a protocol similar to ours^39^. The reason why we could not reproduce their data is currently unknown, but these seemingly disparate results may reflect differences in the cell types used and the environments in which the hamsters were raised. Nevertheless, both our study and that of Lee et al. showed that the administration of senolytic drugs to SARS-CoV-2-infected rodents reduced the levels of inflammatory factors classified as SASP (Fig. 4h), implying that senolysis may be effective in alleviating post-acute COVID-19 syndrome. However, SASP has both harmful and beneficial effects^3,12,13^, and it has recently been reported that the removal of accumulated senescent cells in mice resulted in severe liver dysfunction^40^. In this regard, it is interesting to note that hamsters infected with SARS-CoV-2 showed resistance to superinfection with influenza virus A H1N1 (Supplementary Fig. 3). Thus, it is also tempting to speculate that SARS-CoV-2-induced senescent cells may have some beneficial effects, depending on the biological context. Accordingly, a more rigorous analysis is needed to determine whether senolysis can serve as a preventive measure against post-acute COVID-19 syndrome. Nevertheless, our findings provide valuable new insights into the mechanism of post-acute COVID-19 development^1^ and suggest new possibilities for its control.

## Methods

### Animal experiments

All animal experiments were approved by the Animal Research Committee of the Research Institute for Microbial Diseases, Osaka University. Four-week-old male Syrian hamsters were purchased from SLC Japan. SARS-CoV-2 (5.6 × 10^5^ p.f.u. per 80 μl) infections were performed as previously described^41^. At 3 days after infection, animals were randomly divided into the vehicle (5% DMSO and 4% Tween-20 in PBS) or ABT-263 (100 mg kg^−1^ body weight) group, and intraperitoneally treated for days 3–7 and days 10–13 after infection. On day 14 post-infection, animals were euthanasia and perfused by using 10 U ml^−1^ heparin (Mochida Pharmaceutical, 224122557) containing PBS. Isolated lungs were immediately soaked to RNA later (Sigma, R0901) overnight or 4% PFA for 72 h at 4°C. Ten-week-old female BALB/c mice were purchased from SLC Japan. MA10 (1.0 × 10^4^ p.f.u. per 50 μl) intranasal infection was performed the same way as Syrian hamster. After animals awoke from anesthesia, ABT-263 dissolved in 10% EtOH, 30% polyethylene glycol 400 and 60% Phosal 50 PG were administered orally. The dose of ABT-263 was 100 mg kg^−1^ on days 0–3, 50 mg kg^−1^ on days 5–9 and 50 mg kg^−1^ on days 11–13. Lungs were isolated after euthanasia, soaked to TRIzol and homogenized. Lung RNA isolation was performed using TRIzol according to the manufacturer’s protocol. In Fig. 4f–h, animals that died before day 14 were excluded. In the other experiments, no animals were excluded from the analysis.

### Cell culture

Normal HDFs, TIG-3 cells (Japanese Cancer Research Resources Bank (JCRB), 0506), Vero cells (ATCC, CCL-81), VeroE6/TMPRSS2 cells (JCRB, 1819), HepG2 cells (JCRB, 1054) and Syrian hamster embryo fibroblasts established from day 10 hamster embryos (kindly provided by H. Siomi, Keio University) were cultured in Dulbecco’s modified Eagle’s medium supplemented with 10% fetal bovine serum (MP Biomedicals, 2917354H) and 100 U ml^−1^ penicillin/streptomycin (Sigma, P4333), described as normal culture medium. Normal human bronchial/tracheal epithelial cells, NHBE cells (Lonza, CC-2540) and normal human colonic epithelial cells and HCoEpiCs (ScienCell, 2950) were cultured BEBM basal medium (Lonza, CC-3171) supplemented with the BEBM SingleQuots Kit (Lonza, CC-4175) or colonic epithelial cell medium (CoEpiCM, 2951) following the manufacturer’s protocol. Madin–Darby canine kidney (MDCK) cells (kindly provided T. Watanabe, Osaka University) were maintained in minimal essential medium (Gibco) supplemented with 5% newborn calf serum (Gibco) at 37 °C with 5% CO_2_. MDCK cells were used for plaque assays to titrate H1N1 viruses. For establishing ACE2-HDFs and EGFP-HDFs, retroviral transduction was carried out. Cells were infected with retroviruses encoding ACE2 (in pMarX-hygro) or EGFP/ELuc (in pMarX-puro) and selected by hygromycin (50 μg ml^−1^) or puromycin (100 ng ml^−1^), respectively, for 24 h (ref. ^42^). Cellular senescence was induced by treatment with doxorubicin (DXR) at the following cell densities and DXR concentrations: HDFs and ACE2-HDFs, 8 × 10^3^ cells per cm^2^, DXR 250 ng ml^−1^; NHBE cells, 3 × 10^3^ cells per cm^2^, DXR 150 ng ml^−1^; HCoEpiCs, 8 × 10^3^ cells per cm^2^, DXR 250 ng ml^−1^. After 9 days of treatment with DXR, cells were used as senescent cells. We regularly confirmed the absence of mycoplasma contamination in our cultured cells.

### BOs and BO-derived ALI culture

Bronchial organoids (BOs) were generated as described previously^25^. Briefly, to generate BOs, NHBEs (Lonza, CC-2540) were suspended in 10 mg ml^−1^ cold Matrigel growth factor reduced basement membrane matrix and then cultured with the differentiation medium for 10 days. To generate a BO-derived air–liquid interface (ALI) model, expanding BOs were seeded into Transwell inserts (Corning) in a 24-well plate (3 × 10^4^ cells per well) and then cultured with the differentiation medium for 5 days. The medium was only placed into the bottom chamber to maintain the ALI culture condition.

### Virus preparation

SARS-CoV-2 strains SARS-CoV-2/Hu/DP/Kng/19-020 and SARS-CoV-2/Hu/DP/Kng/19-027 were provided by the Kanagawa Prefectural Institute of Public Health, and SARS-CoV-2 strain hCoV-19/Japan/QHN002/2021 was provided by the National Institute of Infectious Disease. SARS-CoV-2 was isolated from a patient with COVID-19 (GenBank: LC528233.1). All viruses were propagated in Vero cells or VeroE6/TMPRSS2 cells and titers determined by a TCID_50_ or plaque assay as described previously^41^. All experiments, including virus infections, were done in a biosafety level 3 facility at Osaka University and Kyoto University strictly following regulations. SARS-CoV-2/Hu/DP/Kng/19-020 was used in most experiments. SARS-CoV-2/Hu/DP/Kng/19-027 and GenBank: LC528233.1 were used for hBO experiments. hCoV-19/Japan/QHN002/2021 was used for experiments shown in Extended Data Fig. 7 and Syrian hamster experiments. Mouse-adapted SARS-CoV-2 (MA10), established by a reverse genetics method named CPER (circular polymerase extension reaction)^43^, was grown in Vero/TMPRSS2 cells, and the titer was measured using a TCID_50_ assay. A/California/04/2009 (H1N1; CA04), which was kindly provided by Y. Kawaoka at the University of Tokyo, was propagated at 37°C in MDCK cells to make the viral stock. Virus titers were determined by plaque assays in MDCK cells.

### In vitro SARS-CoV-2 infection experiments

ACE2-HDFs, EGFP-HDFs, HDFs, HepG2, NHBE and HCoEpiCs were seeded and cultured 24 h at 37°C, 5% CO_2_. Before virus infection, the medium was replaced with Dulbecco’s modified Eagle’s medium supplemented with 2% fetal bovine serum following two washes in PBS. Then, cells were infected with SARS-CoV-2 (1.0 m.o.i.). At 24 h of infection, the medium was replaced with normal culture medium after two washes in PBS. BO-ALI was infected with SARS-CoV-2 (0.1 m.o.i.). The medium containing SARS-CoV-2 was plated into the top chamber. At 120 min after infection, the medium was removed from the top chamber to reestablish the ALI culture condition. The culture medium in the bottom chamber was replaced with fresh differentiation medium at 1, 2 and 3 days after infection. TNF-α inhibitor (etanercept, 500 μg ml^−1^) was added to SARS-CoV-2-infected hBOs at day 3 after infection.

### Immunofluorescence and immunohistochemistry

Immunofluorescence and immunohistochemistry were performed by using primary antibodies against SARS-CoV-2 spike protein (GeneTex, GTX632604; 1:1,000), SARS-CoV-2 nucleocapsid protein (Sino Biological, 40143-R001; 1:1,000), p16^INK4a^ (Santa Cruz Biotechnology, sc-56330; 1:200), IL-1β (Proteintech, 16806-1-AP; 1:200), IL-8 (Bioss Antibodies, bs-0780R; 1:50), 53BP1 (Santa Cruz Biotechnology, sc-22760; 1:1,000), phospho-p38 (Cell Signaling Technology, 4511; 1:1,000), cleaved caspase-3 (Cell Signaling Technology, 9664; 1:500), ACE2 (R&D Systems, AF933; 1:100), KRT5 (BioLegend, 905903; 1:200) and FoxJ1 (R&D Systems, AF3619; 1:80). The following secondary antibodies were used for immunofluorescence: Alexa Fluor donkey anti-mouse 488 (Invitrogen, A21202; 1:1,000), anti-mouse 555 (Invitrogen, A-31570; 1:1,000), anti-rabbit 568 (Invitrogen, A10042; 1:1,000) and anti-goat 647 (Invitrogen, A-21447; 1:1,000). For immunohistochemistry, we used goat anti-rabbit IgG (Vector Laboratories, BA-1000; 1:1,000). TUNEL staining was performed using the In Situ Cell Death Detection Kit with TMR red (Roche, 12156792910) according to the manufacturer’s protocol. Briefly, TUNEL staining was performed before normal immunofluorescence staining. Fluorescence images were observed using an all-in-one fluorescence microscope (Keyence, BZ-710) or upright microscope (Olympus, BX53). The number of positive signals in the images was calculated in an automated manner using BZ-X 700 analyzer software (Keyence, v1.4.1.1).

### RNA isolation and RT-qPCR

Total RNA was extracted using TRIzol (Thermo Fisher Scientific, 15596026) or the RNeasy mini kit (Qiagen, 74104) according to the manufacturer’s protocol. cDNA was synthesized using a PrimeScript RT reagent kit (Takara Bio, PR047A). The mRNA expression levels of each gene were calculated relative to β-actin or GAPDH expression levels using quantitative qPCR. The PCR primer sequences used are described in Supplementary Table. 1.

### Cell proliferation assay

Live cells were photographed using an Eclipse Ts2R inverted microscope (Nikon) for the indicated time points. Cell numbers were counted using ImageJ (Fiji), and relative cell numbers were calculated.

### Senolysis assay

Normal HDFs and Syrian hamster embryo fibroblasts were rendered senescent by 250 ng ml^−1^ DXR for 9 days or 150 ng ml^−1^ DXR for 6 days, respectively. After senescence induction, senescent cells and control early-passage cells were seeded at 5 × 10^3^ cells per cm^2^ and cultured for 24 h. Then, the senolytic drugs ABT-263 (Selleck, S1001; 1 μM), ARV-825 (Medchem Express, HY-16954; 25 nM), BPTES (Cayman, 19284; 10 μM), dasatinib (LC Laboratories, D-3307; 2 μM) and quercetin (Cayman, 10005169; 20 μM) were added. After 72 h of culture with senolytic drugs, live-cell numbers were counted using trypan blue staining (Invitrogen, T10282), or cells were fixed by 4% paraformaldehyde for 15 min at room temperature. These fixed cell counterstains were utilized DAPI (Dojindo, 340-07971; 1:2,000) for 1 h at room temperature. The photographs were observed using an all-in-one fluorescence microscope (Keyence, BZ-710).

### Induction of cellular senescence using culture supernatants of SARS-CoV-2-infected cells

ACE2-HDFs were infected with SARS-CoV-2 at m.o.i. of 1.0. After 6 days of culture, the supernatant was collected and removed debris by a centrifuge (1,100 × *g*, 15 min). For the preparation of TNF-α knockdown cell supernatant, 3 days after infection, siControl (Santa Cruz Biotechnology, sc-37007) or siTNF-α (Santa Cruz Biotechnology, sc-37216) were transfected using lipofectamine RNAiMAX (Invitrogen, 13778075); at day 6 after infection, the supernatant was collected and debris removed by centrifuge (1,100 × *g*, 15 min). HDFs were seeded at 8.0 × 10^3^ cells in 6-well plates and treated with the supernatant of SARS-CoV-2-infected cells and normal culture medium at a ratio of 1:1. The TNF-α inhibitor etanercept (Pfizer, 46359771; 500 μg ml^−1^), the IL-6R inhibitor tocilizumab (Selleck, A2012; 50 μg ml^−1^), type I IFN neutralization antibody (PBL Assay Science, 39000-1; 1:200) and the p38 MAP kinase inhibitor SB203580 (Selleck, S1076; 10 μM) were administrated at the indicated dose.

### Single-cell RNA sequencing

Single-cell RNA-sequencing data (GSE158127) (ref. ^28^) were reanalyzed by focusing on *CDKN2A* and SASP factor gene expression in epithelial cells using Seurat v3.2.3 and doublet detection by Doublet Finder v2.0.3.

### Immunoblotting

Cells were lysed by RIPA buffer (50 mM Tris-HCl, pH 7.4, 150 mM NaCl, 1 mM EDTA, 0.1% SDS, 1% Triton-X 100 and 1% sodium deoxycholate). Protein sample were suspended 4× Laemmli sample buffer, 50 mM Tris-HCl, pH 6.8, 4% SDS, 10% glycerol, 0.1% bromophenol blue and 1 mM DTT and boiled at 95°C for 5 min in BSL-3. The determination of protein concentration and immunoblotting were performed as previously described^35^. The primary antibodies used were β-actin (Sigma, A5316; 1:1,000), ACE2 (R&D Systems, AF933; 1:1,000), phospho-p38 (Cell Signaling Technology, 4511; 1:1,000) and p38 (Cell Signaling Technology, 9212; 1:1,000). The membranes were then incubated with the secondary antibodies (Cell Signaling Technology, 1:10,000) and visualized with Amersham ECL Prime (GE Healthcare), followed by detection with chemiluminescence using Amersham ImageQuant 800 (Cytiva).

### Statistics

All data were visualized and analyzed in Prism (v.9.1.2). Statistical significance was determined by a two-tailed unpaired Student’s *t* test (Figs. 1a and 2d,e; Extended Data Figs. 1b, 2a,b, 3b, 5a,b and 6a,d,e; and Supplementary Fig. 3c), two-way ANOVA followed by Sidak’s multiple comparison test (Figs. 1e,f, 3a and 4a; Extended Data Figs. 2d, 6b,c,f, 7a,b and 9b,d,e; and Supplementary Fig. 3b), one-way ANOVA followed by Dunnett’s multiple comparison test (Figs. 2h,i, 3b,c and 4c–e,g,h and Extended Data Figs. 4b,c, 5d and 10b) or one-way ANOVA followed by Tukey multiple comparison tests (Extended Data Fig. 8d). *P* values < 0.05 were considered significant. No statistical methods were used to predetermine sample sizes, but our sample sizes were similar to those reported in the previous publications^39^. Data distribution was assumed to be normal, but this was not formally tested. For all experiments, animals and/or cell culture wells were randomly assigned to experimental groups. Data collection and analysis were not performed blind to the conditions of the experiments.

### Reporting Summary

Further information on research design is available in the Nature Research Reporting Summary linked to this article.

## Supplementary information

Supplementary Information Supplementary Figures 1–3 and Table 1 and statistical source data

Reporting Summary

## Data Availability

RNA-sequencing data that support the findings of this study have been deposited in the DNA Data Bank of Japan with the accession number PRJDB11886 (https://ddbj.nig.ac.jp/DRASearch/). All other data supporting the findings of this study are available from the corresponding author upon reasonable request.

## Source data

Source Data Fig. 1 Statistical source data.
Source Data Fig. 2 Unprocessed western blot.
Source Data Fig. 2 Statistical source data.
Source Data Fig. 3 Statistical source data.
Source Data Fig. 4 Statistical source data.
Source Data Extended Data Fig. 1 Unprocessed western blot.
Source Data Extended Data Fig. 1 Statistical source data.
Source Data Extended Data Fig. 2 Statistical source data.
Source Data Extended Data Fig. 3 Statistical source data.
Source Data Extended Data Fig. 4 Statistical source data.
Source Data Extended Data Fig. 5 Statistical source data.
Source Data Extended Data Fig. 6 Statistical source data.
Source Data Extended Data Fig. 7 Statistical source data.
Source Data Extended Data Fig. 8 Statistical source data.
Source Data Extended Data Fig. 9 Statistical source data.
Source Data Extended Data Fig. 10 Statistical source data.
