# Supplementary material for: SARS-CoV-2 infection triggers paracrine senescence and leads to a sustained senescence-associated inflammatory response
Source: Nat Aging. 2022 Jan 25;2(2):115–24. doi: 10.1038/s43587-022-00170-7 (PMC10154207; doi:10.1038/s43587-022-00170-7)
Supplement: Supplementary file 1 — Supplementary Figures 1–3 and Table 1 and statistical source data [file 43587_2022_170_MOESM1_ESM.pdf]

nature portfolio

https://doi.org/10.1038/s43587-022-00170-7

Supplementary information

SARS-CoV-2 infection triggers paracrine senescence and leads to a sustained senescence-associated inflammatory response

In the format provided by the authors and unedited

Supplementary information

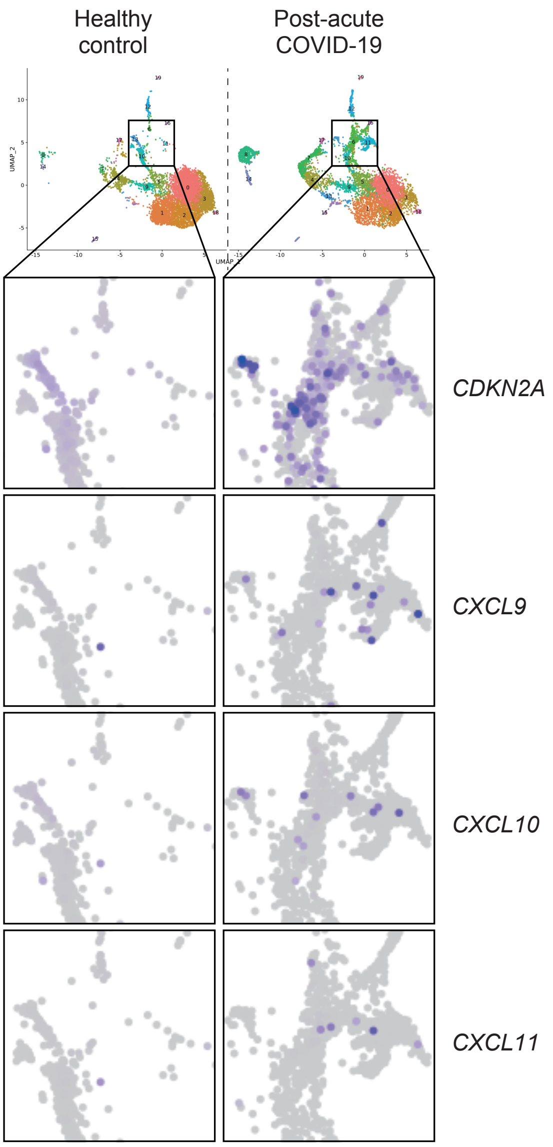

Supplementary Fig. 1

Persistent inflammatory response in patients with COVID-19.

Single-cell RNA transcriptomic analysis of lung tissue from patients with severe COVID-19[28]. Epithelial cells population of Uniform Manifold Approximation and Projection (UMAP) plots was divided into healthy controls (n=2) and COVID-19 patients (n=3), and The distribution of cells expressing the cellular senescence marker (*CDKN2A* gene) and the SASP factor gene, which are also expressed in the Syrian hamster model of SARS-CoV-2 infection.

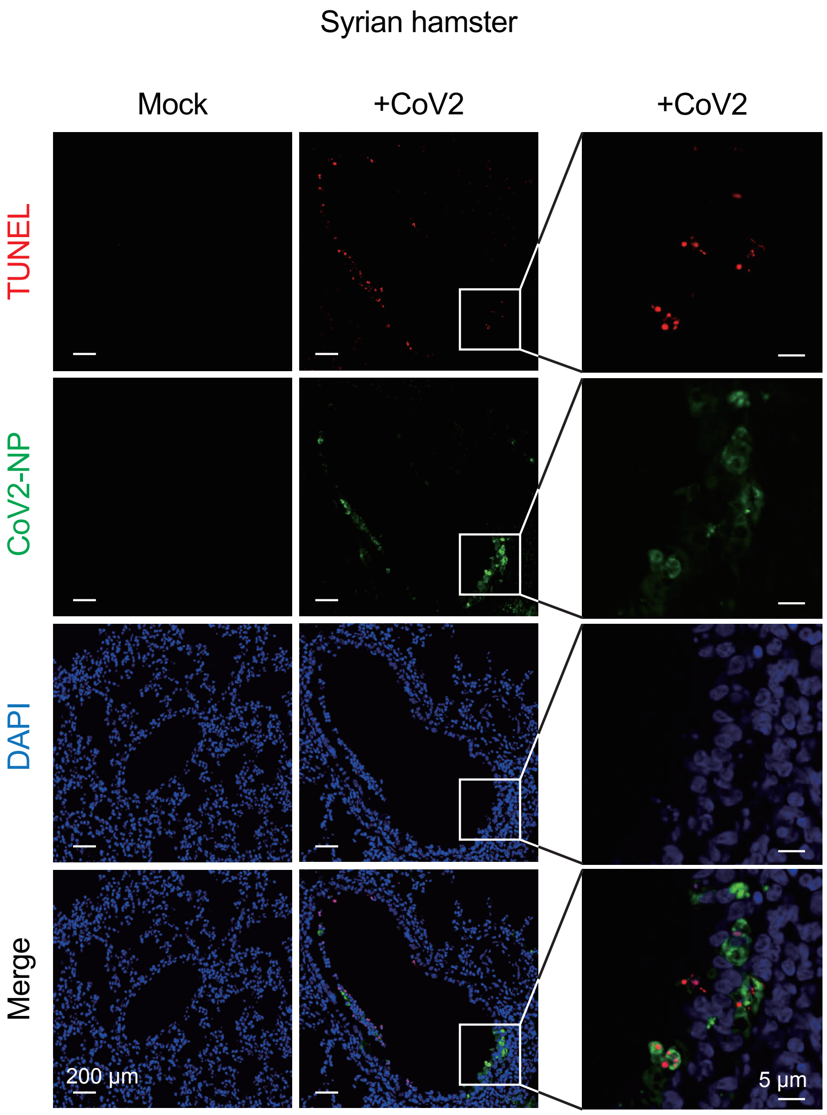

Supplementary Fig. 2

SARS-CoV-2 infection induces cell death.

Syrian hamsters infected Mock (n=3) or SARS-CoV-2 (n=3) were euthanized on days 5 post-infection and lung sections were subjected to TUNEL staining for SARS-CoV-2 N protein (CoV2-NP [green]), TUNEL [red], and DAPI [blue]. Representative images were shown.

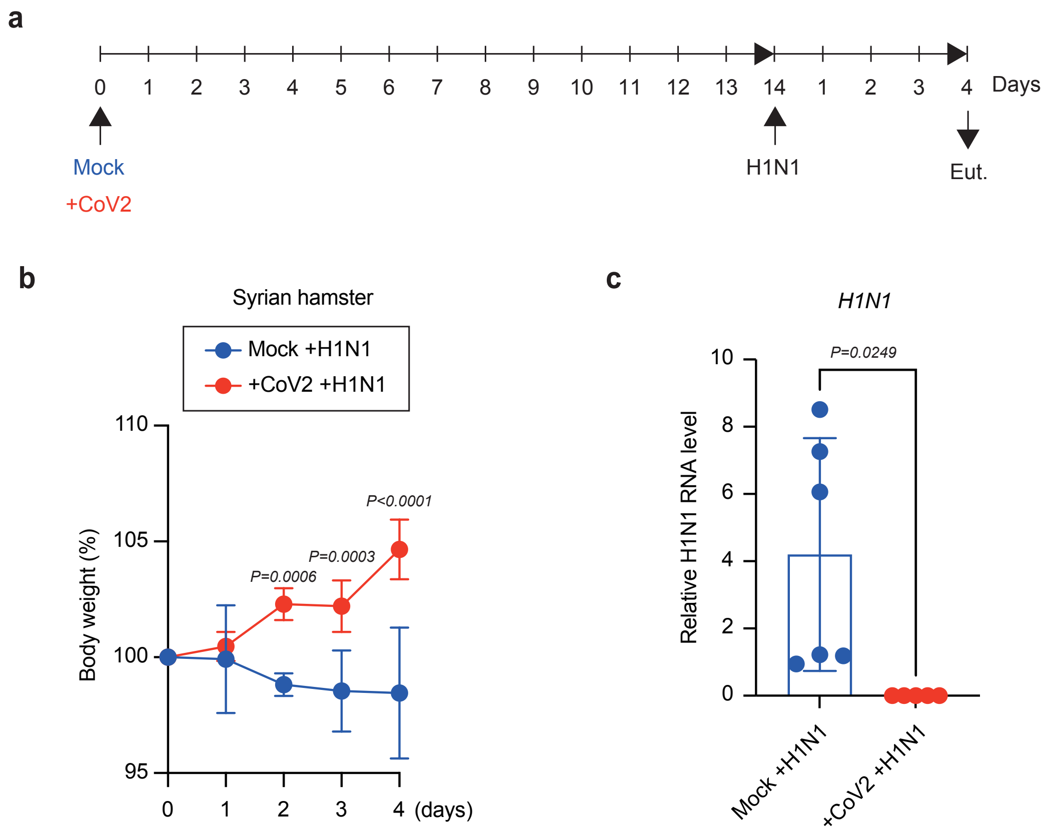

Supplementary Fig. 3

SARS-CoV-2 infection prevents superinfection of influenza virus.

a-c. Syrian hamsters were intranasally inoculated with SARS-CoV-2 (B.1.1.7) 5.6 x 10^5 PFU (in 80 μL), medium (mock). After 14 days post-infection of SARS-CoV-2, mock or SARS-CoV-2 infected hamsters were intranasally inoculated influenza A (H1N1) pdm09 5.6 x 10^5 PFU (in 80 μL). Hamsters infected with mock followed by H1N1 (mock+H1N1; n=6) and SARS-CoV-2 followed by H1N1 (CoV2+H1N1; n=6). The timeline of the experiment was shown (**a**). The body weight was monitored until day 4 post H1N1 infection (**b**). These hamsters were euthanized on day 4 after H1N1 infection, and the expression of H1N1 genomic RNA was analyzed using RT-qPCR. (**c**). For all graphs, error bars indicate mean ± standard deviation (s.d.). Statistical significance was determined two-way ANOVA followed by sidak's multiple comparison test in (b), and with two-tailed unpaired Student's t-test in (c).

Supplementary Table. 1

RT-qPCR Primer Information	
Primer ID	Sequence
human ACE2 Forward	5'- CGAAGCCGAAGACCTGTTCTA -3'
human ACE2 Reverse	5'- GGGCAAGTGTGGACTGTTC -3'
human p16 Forward	5'- ACCAGAGGCAGTAACCATGC -3'
human p16 Reverse	5'- AAGTTTCCCGAGGTTTCTCA -3'
human p21 Forward	5'- AGCGATGGAACTTCGACTTTG -3'
human p21 Reverse	5'- CGAAGTCACCCTCCAGTGGT -3'
human LMNB1 Forward	5'- GATTGCCCAGTTGGAAGCCT -3'
human LMNB1 Reverse	5'- TGGTCTCGTTAATCTCCTCTTCATACA -3'
human TNFa Forward	5'- GCCCCCAGAGGGAAGAGTTCCCCA -3'
human TNFa Reverse	5'- GCT TGAGGGTTTGCTACAACATGGGC -3'
human IL6 Forward	5'- CCAGGAGCCCAGCTATGAAC -3'
human IL6 Reverse	5'- CCCAGGGAGAAGGCAACTG -3'
human IFNb Forward	5'- AAACTCATGAGCAGTCTGCA -3'
human IFNb Reverse	5'- AGGAGATCTTCAGTTTCGGAGG -3'
human IL1B Forward	5'- CTGTCCTGCGTGTTGAAAGA -3'
human IL1B Reverse	5'- TTGGGTAATTTTTGGGATCTACA -3'
human IL8 Forward	5'- AAGGAAAACTGGGTGCAGAG -3'
human IL8 Reverse	5'- ATTGCATCTGGCAACCCTAC -3'
human β-actin Forward	5'- TGGATCAGCAAGCAGGAGTATG -3'
human β-actin Reverse	5'- GCATTTGCGGTGGACGAT -3'
human GAPDH Forward	5'- CAACTACATGGTTTACATGTTC -3'
human GAPDH Reverse	5'- GCCAGTGGACTCCACGAC -3'
syrian hamster p16 Forward	5'- AGAGGTTCGGGCTTTGCT -3'
syrian hamster p16 Reverse	5'- CTACTTGGGTGTTGCCCATC -3'
syrian hamster p21 Forward	5'- TTGTTCGGAGGCACCACCAT -3'
syrian hamster p21 Reverse	5'- TCGAAGTTCCACCGTTCTCG -3'
syrian hamster Ccl17 Forward	5'- GTGCTGCCTGGAGATCTTCA -3'
syrian hamster Ccl17 Reverse	5'- TGGCATCCCTGGGACACT -3'
syrian hamster Ifng Forward	5'- TGTTGCTCTGCCTCACTCAGG -3'
syrian hamster Ifng Reverse	5'- AAGACGAGGTCCCCTCCATTC -3'
syrian hamster Cxcl9 Forward	5'- ACTCTGCTCTGCCATGAAGT -3'
syrian hamster Cxcl9 Reverse	5'- TTCCTTATGACTAGGGTTCCTTGAA -3'
syrian hamster Cxcl10 Forward	5'- GCCATTCATCCACAGTTGACA -3'
syrian hamster Cxcl10 Reverse	5'- CATGGTGCTGACAGTGGAGTCT -3'
syrian hamster Cxcl11 Forward	5'- CCGCCTCATACGGGAAATGT -3'
syrian hamster Cxcl11 Reverse	5'- AAGACAGAAGGTTGGGCTCG -3'
syrian hamster Gapdh Forward	5'- TTGTTGCCATCAATGACCCCTT -3'
syrian hamster Gapdh Reverse	5'- CGTTCTCAGCCTTGACTGTGCCTT -3'
mouse β-actin Forward	5'- GATGACCCAGATCATGTTTGA -3'
mouse β-actin Reverse	5'- GGAGAGCATAGCCCTCGTAG -3'
mouse Gapdh Forward	5'- CAACTACATGGTCTACATGTTC -3'
mouse Gapdh Reverse	5'- CGCCAGTAGACTCCACGAC -3'
mouse p16Ink4a Forward	5'- GAACTCTTTCGGTCGTACCC -3'
mouse p16Ink4a Reverse	5'- CGAATCTGCACCGTAGTTGA -3'
mouse p19Arf Forward	5'- GCCGCACCGGAATCCT -3'
mouse p19Arf Reverse	5'- TTGAGCAGAAGAGCTGCTACGT -3'
mouse Ifnb Forward	5'- CAGCTCCAAGAAAGGACGAAC -3'
mouse Ifnb Reverse	5'- GGCAGTGTAACTCTTCTGCAT -3'
mouse Ifng Forward	5'- ATGAACGCTACACACTGCATC -3'
mouse Ifng Reverse	5'- CCATCCTTTTGCCAGTTCCTC -3'
mouse Cxcl9 Forward	5'- CCTAGTGATAAGGAATGCACGATG -3'
mouse Cxcl9 Reverse	5'- CTAGGCAGGTTTGATCTCCGTTC -3'
mouse Cxcl10 Forward	5'- ATCATCCCTGCGAGCCTATCCT -3'
mouse Cxcl10 Reverse	5'- GACCTTTTTTGGCTAAACGCTTTC -3'
mouse Mmp10 Forward	5'- TGCTGCCTATGAGGCTCACAAC -3'
mouse Mmp10 Reverse	5'- GGAGGAAAACCGAGAGTGTGGA -3'
SARS-CoV-2 N Forward	5'- TTACAAACATTGGCCGCAAA -3'
SARS-CoV-2 N Reverse	5'- GCGCGACATTCCGAAGAA -3'
SARS-CoV subgenomic N Forward	5'- CCAGGTAACAAACCAACCAACTTTCG -3'
SARS-CoV subgenomic N Reverse	5'- GGTTACTGCCAGTTGAATCTGAGG -3'
H1N1 pdm09 Forward	5'- GGAGCAAAAAGCTTCTACAA -3'
H1N1 pdm09 Reverse	5'- ACTTTGTTGGTCAGCACTA -3'

Sauce Data of Supplementary figure. 3

S. Fig. 3b					
Body weght (%)					
days	0	1	2	3	4
Mock+H1N1	100	98.3298539	99.0605428	100	99.3736952
	100	99.7918835	98.855359	96.2539022	96.0457856
	100	99.5014955	97.9062812	97.4077767	97.108674
	100	104.314995	98.8133765	97.3031284	95.0377562
	100	99.9016716	98.9183874	100	101.966568
	100	97.6626016	99.3902439	100.304878	101.219512
CoV2+H1N1	100	101.117886	101.727642	101.626016	103.556911
	100	100.209424	102.513089	104.293194	106.910995
	100	100.432432	102.378378	101.513514	103.459459
	100	99.7402597	102.467532	101.688312	105.324675
	100	100	101.354402	102.708804	104.288939
	100	101.317365	103.353293	101.437126	104.431138
Adjusted P Value	>0.9999	0.9711	0.0006	0.0003	<0.0001
Two-way ANOVA Šídák's multiple comparisons test					

S. Fig. 3c	
Relative (to GAPDH) H1N1 genomic RNA level	
Mock+H1N1	CoV2+H1N1
7.260153243	2.0215E-08
8.51496146	9.10938E-09
0.946057647	7.60713E-09
1.189207115	5.41647E-09
6.062866266	1.43936E-08
1.222640278	ND
Unpaired t test	Two-tailed
P value	0.0249
