# Supplementary material for: SARS-CoV-2 infection triggers paracrine senescence and leads to a sustained senescence-associated inflammatory response
Source: Nat Aging. 2022 Jan 25;2(2):115–24. doi: 10.1038/s43587-022-00170-7 (PMC10154207; doi:10.1038/s43587-022-00170-7)
Supplement: Supplementary file 2 — Reporting Summary [file 43587_2022_170_MOESM2_ESM.pdf]

nature portfolio

Corresponding author(s): Eiji Hara

Last updated by author(s): Dec 27, 2021

Reporting Summary

Nature Portfolio wishes to improve the reproducibility of the work that we publish. This form provides structure for consistency and transparency in reporting. For further information on Nature Portfolio policies, see our Editorial Policies and the Editorial Policy Checklist.

Statistics

For all statistical analyses, confirm that the following items are present in the figure legend, table legend, main text, or Methods section.

n/a	Confirmed	
☐	☒	The exact sample size (*n*) for each experimental group/condition, given as a discrete number and unit of measurement
☐	☒	A statement on whether measurements were taken from distinct samples or whether the same sample was measured repeatedly
☐	☒	The statistical test(s) used AND whether they are one- or two-sided<br>*Only common tests should be described solely by name; describe more complex techniques in the Methods section.*
☒	☐	A description of all covariates tested
☒	☐	A description of any assumptions or corrections, such as tests of normality and adjustment for multiple comparisons
☐	☒	A full description of the statistical parameters including central tendency (e.g. means) or other basic estimates (e.g. regression coefficient) AND variation (e.g. standard deviation) or associated estimates of uncertainty (e.g. confidence intervals)
☐	☒	For null hypothesis testing, the test statistic (e.g. *F*, *t*, *r*) with confidence intervals, effect sizes, degrees of freedom and *P* value noted<br>*Give P values as exact values whenever suitable.*
☒	☐	For Bayesian analysis, information on the choice of priors and Markov chain Monte Carlo settings
☒	☐	For hierarchical and complex designs, identification of the appropriate level for tests and full reporting of outcomes
☒	☐	Estimates of effect sizes (e.g. Cohen's *d*, Pearson's *r*), indicating how they were calculated

Our web collection on statistics for biologists contains articles on many of the points above.

Software and code

Policy information about availability of computer code

Data collection	Microscopic data: Fluorescence Microscope BZ-X710 (KEYENCE)<br>Quantitative real-time PCR data: Thermal Cycler Dice Real Time System III (Takara)<br>Immunoblotting data: Amersham ImageQuant800 (cytiva)<br>Histological data: BX53 Upright Microscope (OLYMPUS)
Data analysis	Immunoblotting analysis: ImageJ (version 2.3.0)<br>Immunofluorescence staining analysis: BZ-X analyzer software (version 1.4.1.1)<br>Single cell RNA sequence analysis: Seurat (version 3.2.3), Doublet Finder (version 2.0.3)<br>Statistical analysis: GraphPad Prism 9, R (version 9.2.0)

For manuscripts utilizing custom algorithms or software that are central to the research but not yet described in published literature, software must be made available to editors and reviewers. We strongly encourage code deposition in a community repository (e.g. GitHub). See the Nature Portfolio guidelines for submitting code & software for further information.

Data

Policy information about availability of data

All manuscripts must include a data availability statement. This statement should provide the following information, where applicable:

- Accession codes, unique identifiers, or web links for publicly available datasets
- A description of any restrictions on data availability
- For clinical datasets or third party data, please ensure that the statement adheres to our policy

RNA sequence data that support the findings of this study have been deposited in the DNA Data Bank of Japan (DDBJ) with the accession number PRJDB11886 (https://ddbj.nig.ac.jp/DRASearch/). All other data supporting the findings of this study are available from the corresponding author upon reasonable request.

Field-specific reporting

Please select the one below that is the best fit for your research. If you are not sure, read the appropriate sections before making your selection.

☒ Life sciences ☐ Behavioural & social sciences ☐ Ecological, evolutionary & environmental sciences

For a reference copy of the document with all sections, see nature.com/documents/nr-reporting-summary-flat.pdf

Life sciences study design

All studies must disclose on these points even when the disclosure is negative.

Sample size	The sample size used in this study was determined based on the expense of data collection, and the need to have sufficient statistical power.
Data exclusions	In Fig. 4 f to h, individuals who died before day 14 were excluded. For the other experiments, no data was excluded from the analysis.
Replication	All data presented were obtained from three or two independent experiments with similar outcomes. (see Figure legends)
Randomization	For all experiments, animals and/or cell culture wells were randomly assigned to experimental groups.
Blinding	Data collection and analysis were not performed blind to the conditions of the experiments, because experiments were performed and analyzed by the same researchers. However, all experiments and analysis were performed objective and unbiased.

Reporting for specific materials, systems and methods

We require information from authors about some types of materials, experimental systems and methods used in many studies. Here, indicate whether each material, system or method listed is relevant to your study. If you are not sure if a list item applies to your research, read the appropriate section before selecting a response.

Materials & experimental systems

n/a	Involved in the study	
☐	☒	Antibodies
☐	☒	Eukaryotic cell lines
☒	☐	Palaeontology and archaeology
☐	☒	Animals and other organisms
☒	☐	Human research participants
☒	☐	Clinical data
☒	☐	Dual use research of concern

Methods

n/a	Involved in the study	
☒	☐	ChIP-seq
☒	☐	Flow cytometry
☒	☐	MRI-based neuroimaging

Antibodies

Antibodies used	SARS-CoV-2 spike protein (GeneTex, GTX632604, clone 1A9), SARS-CoV-2 Nucleocapsid protein (Sino Biological, 40143-R001, clone 001), p16INK4a (Santa Cruz, sc-56330, clone JC8), IL1β (proteintech, 16806-1-AP), IL8 (Bioss Antibodies, bs-0780R), 53BP1 (Santa Crus, sc-22760, clone H-300), phospho-p38 (Cell Signaling Tecnology, 4511, clone D3F9), cleaved caspase 3 (Cell Signaling Tecnology, 9664, clone 5A1E), ACE2 (R&D, AF933), KRT5 (BioLegend, 905903), FoxJ1 (R&D, AF3619), β-actin (Sigma, A5316, clone AC-74), p38 (Cell Signaling Tecnology, 9212), Type I IFN Neutralization Antibody Mixture (pbl assay science, 39000-1)<br>Goat anti-rabbit IgG (Vector Laboratories, BA-1000), Donkey anti-mouse IgG Alexa Fluor 488 (Invitrogen, A21202), Donkey anti-rabbit IgG Alexa Fluor 568 (Invitrogen, A10042), Donkey anti-mouse IgG Alexa Fluor Plus 555 (Invitrogen, A-31570), Donkey anti-goat IgG Alexa Fluor Plus 647 (Invitrogen, A-21447)
Validation	All antibodies used in this study were commercially available antibodies and were validated by companies. Data sheet is available from the web links as described below.

SARS-CoV-2 spike protein; https://www.genetex.com/Product/Detail/SARS-CoV-SARS-CoV-2-COVID-19-spike-antibody-1A9/GTX632604
SARS-CoV-2 Nucleocapsid protein : https://jp.sinobiological.com/antibodies/cov-nucleocapsid-40143-r001
p16INK4a: https://datasheets.scbt.com/sc-56330.pdf
L1β : https://www.ptglab.co.jp/products/IL1B-Antibody-16806-1-AP.htm
IL8 : https://www.biossantibodies.com/datasheets/bs-0780R
53BP1: https://datasheets.scbt.com/sc-22760.pdf
phospho-p38 : https://www.cellsignal.jp/datasheet.jsp?productId=4511&images=0
Cleaved caspase 3 : https://www.cellsignal.jp/datasheet.jsp?productId=9664&images=0
ACE2 : https://www.rndsystems.com/products/human-mouse-rat-hamster-ace-2-antibody_af933
KRT5: https://www.biolegend.com/ja-jp/products/purified-anti-keratin-5-polyclonal-chicken-antibody-15091?GroupID=GROUP26
FoxJ1: https://www.rndsystems.com/products/human-foxj1-antibody_af3619
β-actin : https://www.sigmaaldrich.com/catalog/product/sigma/a5316?lang=ja&region=JP
p-38 : https://www.cellsignal.jp/datasheet.jsp?productId=9212&images=1
Type I IFN Neutralization Antibody Mixture : https://www.pblassaysci.com/antibodies/human-type-1-ifn-neutralizing-antibody-mixture-39000#specifications
Goat anti-rabbit IgG : https://vectorlabs.com/biotinylated-goat-anti-rabbit-igg-antibody.html
Donkey anti-mouse IgG Alexa Fluor 488 : https://www.thermofisher.com/antibody/product/Donkey-anti-Mouse-IgG-H-L-Highly-Cross-Adsorbed-Secondary-Antibody-Polyclonal/A-21202
Donkey anti-rabbit IgG Alexa Fluor 568 : https://www.thermofisher.com/antibody/product/Donkey-anti-Rabbit-IgG-H-L-Highly-Cross-Adsorbed-Secondary-Antibody-Polyclonal/A10042
Donkey anti-mouse IgG Alexa Fluor Plus 555 : https://www.thermofisher.com/antibody/product/Donkey-anti-Mouse-IgG-H-L-Highly-Cross-Adsorbed-Secondary-Antibody-Polyclonal/A-31570
Donkey anti-goat IgG Alexa Fluor Plus 647 : https://www.thermofisher.com/antibody/product/Donkey-anti-Goat-IgG-H-L-Cross-Adsorbed-Secondary-Antibody-Polyclonal/A-21447

Eukaryotic cell lines

Policy information about cell lines

Cell line source(s)
TIG-3 cells (human diploid fibroblast: HDFs), HepG2 cells, and VeroE6/TMPRSS2 cells were obtained from Japanese Cancer Research Resources Bank (JCRB). Vero cells were obtained from ATCC. NHBE cells were obtained from Lonza. HCoEpiC cells were obtained from ScienCell. The human bronchial organoid was supplied by Kyoto University. Syrian hamster embryo fibrblasts was supplied by Keio University. MDCK cells was supplied by Tokyo University.

Authentication
Authentication of human bronchial organoid was described in reference article 25. Other cells were obtained from public bioresources bank or Company and were not authenticated by ourselves.

Mycoplasma contamination
We have confirmed that there were not mycoplasma contamination in our tissue culture cells and were stated in "Cell culture section" of the METHOD page.

Commonly misidentified lines
(See ICLAC register)
No commonly misidentified cell lines were used in this study.

Animals and other organisms

Policy information about studies involving animals; ARRIVE guidelines recommended for reporting animal research

Laboratory animals
Four weeks old male Syrian hamsters and ten weeks old female Balb/c mice were purchased from SLC Japan. The animals were maintained at 23°C±2°C, humidity 55%±15%, on a 12-h light-dark cycle, and fed normal diet (CE-2 from CLEA Japan Inc., sterilized 20 kGy radiation exposure.)

Wild animals
The study did not involve wild animals.

Field-collected samples
The study did not involve samples collected from the field.

Ethics oversight
All mouse experiments were approved by the Animal Research Committee of Research Institute for Microbial Diseases, Osaka University.

Note that full information on the approval of the study protocol must also be provided in the manuscript.
