# Supplementary figures and images for: SARS-CoV-2 infection triggers paracrine senescence and leads to a sustained senescence-associated inflammatory response
Source: Nat Aging. 2022 Jan 25;2(2):115–24. doi: 10.1038/s43587-022-00170-7 (PMC10154207; doi:10.1038/s43587-022-00170-7)

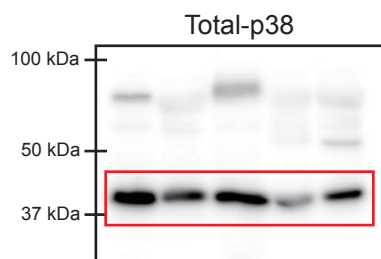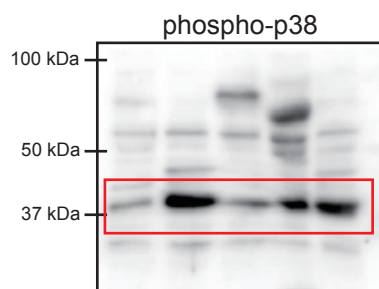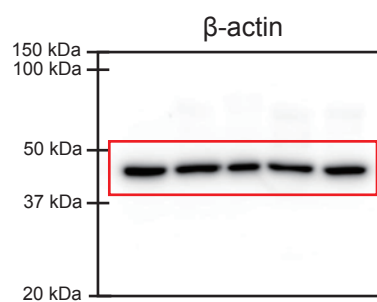

Supplement: Source Data Fig. 2 — Unprocessed western blot. [file 43587_2022_170_MOESM4_ESM.pdf]

ACE2

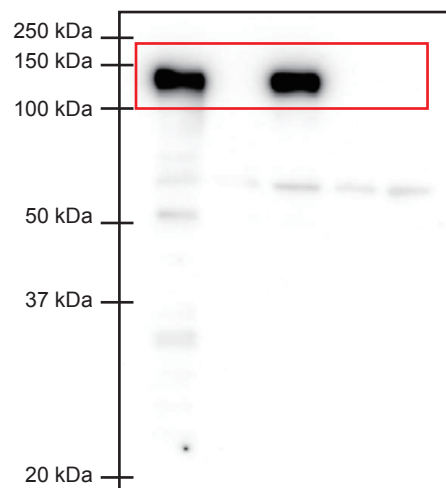

$\beta$ -actin

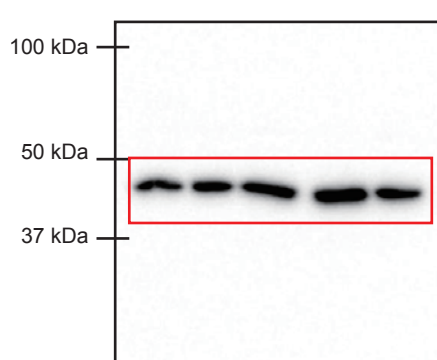

Supplement: Source Data Extended Data Fig. 1 — Unprocessed western blot. [file 43587_2022_170_MOESM8_ESM.pdf]
